# Microbial Communities of Seawater and Coastal Soil of Russian Arctic Region and Their Potential for Bioremediation from Hydrocarbon Pollutants

**DOI:** 10.3390/microorganisms10081490

**Published:** 2022-07-24

**Authors:** Ekaterina M. Semenova, Tamara L. Babich, Diyana S. Sokolova, Alexey P. Ershov, Yeva I. Raievska, Salimat K. Bidzhieva, Alexey L. Stepanov, Maria V. Korneykova, Vladimir A. Myazin, Tamara N. Nazina

**Affiliations:** 1 Winogradsky Institute of Microbiology, Research Center of Biotechnology of the Russian Academy of Sciences, 119071 Moscow, Russia; semenova_inmi@mail.ru (E.M.S.); microb101@yandex.ru (T.L.B.); sokolovadiyana@gmail.com (D.S.S.); e.alexey.mail@inmi.ru (A.P.E.); raievska.yeva@mail.ru (Y.I.R.); salima.bidjieva@gmail.com (S.K.B.); 2 Soil Department, Moscow State University, 119991 Moscow, Russia; stepanov_aleksey@mail.ru; 3 Institute of North Industrial Ecology Problems—Subdivision of the Federal Research Centre “Kola Science Centre of Russian Academy of Science”, 184209 Apatity, Russia; myazinv@mail.ru; 4 Agrarian and Technological Institute, Peoples’ Friendship University of Russia (RUDN University), 117198 Moscow, Russia

**Keywords:** arctic seawater, soil, microorganisms, 16S rRNA gene sequencing, phylogeny, functional diversity, iVikodak, KEGG, hydrocarbon degradation, psychrophiles

## Abstract

The development of Arctic regions leads to pollution of marine and coastal environments with oil and petroleum products. The purpose of this work was to determine the diversity of microbial communities in seawater, as well as in littoral and coastal soil, and the potential ability of their members to degrade hydrocarbons degradation and to isolate oil-degrading bacteria. Using high-throughput sequencing of the V4 region of the 16S rRNA gene, the dominance of bacteria in polar communities was shown, the proportion of archaea did not exceed 2% (of the total number of sequences in the libraries). Archaea inhabiting the seawater belonged to the genera *Nitrosopumilus* and *Nitrosoarchaeum* and to the *Nitrososphaeraceae* family. In the polluted samples, members of the Gammaproteobacteria, Alphaproteobacteria, and Actinomycetes classes predominated; bacteria of the classes Bacteroidia, Clostridia, Acidimicrobiia, Planctomycetia, and Deltaproteobacteria were less represented. Using the iVikodak program and KEGG database, the potential functional characteristics of the studied prokaryotic communities were predicted. Bacteria were potentially involved in nitrogen and sulfur cycles, in degradation of benzoate, terephthalate, fatty acids, and alkanes. A total of 19 strains of bacteria of the genera *Pseudomonas*, *Aeromonas*, *Oceanisphaera*, *Shewanella*, *Paeniglutamicibacter*, and *Rhodococcus* were isolated from the studied samples. Among them were psychrotolerant and psychrophilic bacteria growing in seawater and utilizing crude oil, diesel fuel, and motor oils. The data obtained suggest that the studied microbial communities could participate in the removal of hydrocarbons from arctic seawater and coastal soils and suggested the possibility of the application of the isolates for the bioaugmentation of oil-contaminated polar environments.

## 1. Introduction

In recent years, the development of Arctic Regions has led to an increase in the amount of fuel used, delivered by sea, which in turn is accompanied by the man-made pollution of marine waters and coastal soils. Offshore oil and gas production also carries additional environmental risks for marine ecosystems. Despite the fact that the Arctic Council countries have approved a course for the introduction of renewable energy sources in the region, diesel fuel is currently one of the most used fuels here [1]. The emergence of environmental problems necessitates the search for methods of cleaning polluted ecosystems. Climate features impede the use of physical, thermal, and chemical purification methods [2]. In this regard, there has been a significant increase in interest in environmentally safe and economical biological methods for the remediation of Arctic soils and water basins from oil pollution [3,4,5]. Biostimulation and bioaugmentation biotechnologies are widely used for the bioremediation of polluted habitats [2,6]. The biostimulation method is based on the activation of indigenous microorganisms by additional aeration and the introduction of minerals and organic nutrients, whereas bioaugmentation involves the introduction of exogenous microorganisms to increase the activity of the target group [2,6,7]. Bioaugmentation helps to achieve a greater degree of hydrocarbon degradation in a short period and is used when the natural microorganisms cannot cope with pollution. The success of its application depends on the ability of the introduced bacteria to be active under the specified conditions. The argument in favor of the use of biostimulation is that natural microorganisms are initially well adapted to the conditions of their environment. However, under Arctic conditions, the application of bioremediation methods faces additional difficulties [4,7,8]. Microorganisms living in high-latitude Arctic and Antarctic regions, along with low temperatures, can simultaneously experience several stressors. Soil microbial communities face a shortage of nitrogen and phosphorus, slightly acidic conditions, and low rainfall in summer [4,9,10]. The microbiota of Arctic marine areas lives in a high salinity of sea water, in slightly alkaline conditions and under a varying content of dissolved oxygen [3]. In this regard, the development of biological preparations requires the search for microorganisms capable of using different components of oil and petroleum products at a low temperature and a high salinity of the medium.

Complex hydrocarbon compounds in nature are decomposed in ascending order of their bioavailability: linear alkanes; branched alkanes; low molecular weight alkylaromatic compounds; monocyclic aromatic hydrocarbons; cyclic alkanes; polycyclic aromatic hydrocarbons (PAHs); high molecular weight asphaltenes [11,12,13]. To ensure the entry of a water-insoluble or poorly soluble hydrocarbon molecule into the cell, microorganisms produce surfactants (surface active compounds) associated with the cell or released into the medium, facilitating the contact of cells with a hydrophobic substrate. A decrease in the surface tension of the culture medium against the air and the interfacial tension of the medium at the boundary with the hydrocarbon is an indirect indication of surfactant formation and the ability of the microorganism to degrade hydrocarbons [14,15,16].

Currently, molecular biological methods are widely used to study the composition and metabolic capabilities of microbial communities, including high-throughput sequencing as well as metagenomic and proteomic analyses [17,18,19]. The composition of the microbial communities of Arctic habitats and their metabolic potential has been poorly studied [20]. Arctic microbial communities, especially those inhabiting the soils and waters of the northern latitudes of Russia are poorly represented in the GenBank database [9]. Although the results of molecular studies allow us to predict the metabolic potential of the microbial community as a whole, the isolation and study of the physiology and genomic characteristics of pure cultures of hydrocarbon-oxidizing bacteria allows us to clarify the conclusions made and select promising bacteria for bioremediation of polluted soils and water areas.

The purpose of this work was to determine the phylogenetic diversity of prokaryotes in seawater, littoral and coastal soils sampled in polluted areas of the Murmansk region (Russia), and the potential ability of microorganisms to participate in the biogeochemical cycles of carbon, sulfur, and nitrogen, as well as the isolation of pure bacterial cultures that degrade oil and petroleum products at a low temperature.

## 2. Materials and Methods

### 2.1. Objects of Investigation and Sampling Procedures

The studied samples of seawater and coastal soil were collected in the Murmansk region (69°16′16.1″ N; 29°27′37.9″ E) belonging to the Atlantic–Arctic region of the European part of Russia. The average winter air temperatures in January and in the warmest summer month (July) are −11 and +12 °C, respectively. The subjects of the study were eight samples, including seawater of the Kola Bay of the Barents Sea (1_M21, 2_M21); soil samples (=sandy mud) from the littoral zone flooded by the tide twice a day (3_M21, 6_M21, 10_M21); and coastal soil samples located further from the water’s edge and flooded only during storms (7_M21, 9_M21). The samples were taken in November 2020 on the territory of the settlements of Belokamenka, Roslyakovo, Kola, and Pechenga (Figure S1). The settlements of Belokamenka and Roslyakovo are located in the middle knee of the Kola Bay on the northwestern and southeastern shores, respectively; the distance between them in a straight line across the bay is 3.2 km. When traveling from west to east, there is a significant increase in the depth of the bay. To the south is the ice-free port of Murmansk. Kola is located in the southern knee of the Kola Bay (the mouth of the Kola River). The confluence of the Tuloma and Kola Rivers into the bay leads to a decrease in the salinity of the water compared to the waters of the Barents Sea. Samples from the littoral zone were also taken in the southern part of the Pechenga Bay in the village of Pechenga. All sites under study are subject to anthropogenic impact to one degree or another. According to the Ministry of Natural Resources, Ecology and Fisheries of the Murmansk Region, in 2020, an increased content of nickel, copper, manganese, dithiophosphate, iron, zinc and organic substances (according to chemical oxygen consumption) was observed throughout the Pechenga River, the average annual value of nickel and copper in the waters was noted at the level of 4–5 and 6–8 maximum permissible concentrations (MPC), respectively (http://mpr.gov-murman.ru (accessed on 21 June 2022)). Petroleum products were present in the waters of the Kola Bay, both in dissolved form and in the form of a film on the water surface. The average annual content of petroleum products was noted at the level of 1 MPC.

Water from the surface of the bay (depth 0–30 cm) was collected into sterile plastic bottles. Soil samples from the littoral zone and coastal soil were collected from a depth of 10 cm by the envelope method in sterile plastic containers. The samples were stored at a temperature of 4 °C.

### 2.2. Isolation of DNA from the Studied Samples and Pure Cultures and 16S rRNA Gene Sequencing

Averaged samples of soils (10 g) and water (1 l) were used to isolate total DNA using the Fast DNA Spin Kit (MPBio, Solon, Ohio USA) in accordance with the manufacturer’s instructions. DNA for analysis of the composition of microbial communities was isolated from 7 of 8 samples. The DNA of pure cultures was isolated by the Pure Link Microbiome DNA Purification KIT (Thermo Fisher Scientific, Waltham, MA, USA). The 16S rRNA genes of pure cultures were amplified using DNA samples and universal primers 8-27f and 1492r [21]. Sequencing of PCR products of the 16S rRNA gene fragments of pure cultures was performed using a 3730 DNA Analyzer and the BigDye ^®^ Terminator v3.1 Cycle Sequencing Kits (Applied Biosystems, Waltham, MA, USA) in accordance with the manufacturer’s recommendations.

Assembly and editing of the nucleotide sequences were carried out using Bioedit (http://jwbrown.mbio.ncsu.edu/BioEdit/bioedit.html (accessed on 9 March 2017)). The obtained sequences were compared with genes of the reference prokaryotes available in GenBank database using the BLAST algorithm (NCBI server, www.ncbi.nlm.nih.gov/blast/ (accessed on 17 March 2022)).

To obtain libraries of the 16S rRNA gene of the studied microbial communities, the V4 hypervariable region of this gene was amplified based on double barcoding. The V4 region of the 16S rRNA gene was amplified using a direct primer (5′-CAAGCAGAAGACGGCATACGAGATGTGACTGGAGTTCAGACGTGTGCTCTTCCGATCT XXXXXX ZZZZ GTGBCAGCMGCCGCGGTAA-3′) and reverse primer (5′-AATGATACGGCGACCACCGAGATCTACACTCTTTCCCTACACGACGCTCTTCCGATCT XXXXXX ZZZZ GACTACNVGGGTMTCTAATCC-3′) [22]. Sequencing was performed using MiSeq platform (Illumina, San Diego, CA, USA) and MiSeqReagentKitv3 reagent kit (Illumina, San Diego, CA, USA) in accordance with the manufacturer’s recommendations.

### 2.3. Bioinformatic Analysis

The obtained fragments with a length of 250 bp were subjected to quality control using UPARSE software [23], and then qualified reads were grouped to create operational taxonomic units (OTUs) with a 97% similarity level using USEARCH software [24]. For a representative sequence, a taxonomic position was determined for each OTU using the SILVA database (SILVA, https://www.arb-silva.de/aligner/, v. 1.2.11, accessed on 29 September 2021, SILVA reference database release 138.1) [25]. Prokaryotic sequences with ≥97% similarity, combined into OTUs and identified using the SILVA online resource, were used as input in Global Mapper module iVikodak software (https://web.rniapps.net/iVikodak/global.php (accessed on 18 July 2022)) package for prediction of the functional characteristics of prokaryotic communities [26]. The Kyoto Encyclopedia of Genes and Genomes (KEGG) database (https://www.genome.jp/kegg/mapper/color.html (accessed on 18 July 2022)) was used to obtain the functional profiles and individual profiles of the enzymes of nitrogen, sulfur, benzoate, and methane metabolism and degradation of polycyclic aromatic hydrocarbons and fatty acids. Heatmaps of the functional profiles predicted for the communities were constructed using the ClustVis Internet resource (http://biit.cs.ut.ee/clustvis/, (accessed on 8 April 2021)). The Venn diagram was constructed using the Venny online resource (http://bioinfogp.cnb.csic.es/tools/venny/ (accessed on 8 April 2021)).

### 2.4. Composition of Nutrient Media, Conditions of Cultivation and Isolation of Microorganisms

#### 2.4.1. Sample Preparation

To quantify and isolate the microorganisms from soil samples, a suspension containing an average sample weight of 10 g and 90 mL of sterile tap water was obtained. The suspension was stirred using an Unimax 1010 shaker (Heidolph Instruments, Schwabach, Germany) at 120 rpm for 10 min, and then left for 5 min to precipitate the solid phase. The obtained soil suspensions and seawater samples were used for inoculating the liquid selective nutrient media by the method of serial ten-fold dilutions. The results were evaluated by the most probable number method according to the McCredy table [27].

#### 2.4.2. Media Compositions

The number of aerobic organotrophic bacteria (AOB) was evaluated using PC medium containing (per liter distilled water): 1.0 g glucose, 5.0 g tryptone, 2.5 g yeast extract, 20.0 g NaCl, pH 7.0–7.2. The number of hydrocarbon-oxidizing bacteria (HOB) was determined using mineral medium (MM) containing (per liter distilled water): 1.5 g K_2_HPO_4_, 0.75 g KH_2_PO_4_, 1.0 g NH_4_Cl, 20.0 g NaCl, 0.1 g KCl, 0.1 g MgSO_4_·7H_2_O, 0.02 g CaCl_2_·2H_2_O, pH 7.0. A mixture of C_14_-C_17_ *n*-alkanes (2.0 mL·L^−1^) was added to the medium as a carbon source. The anaerobic Hungate’s technique [28] was used to prepare media for anaerobic bacteria. The number of fermenting microorganisms was estimated using the medium containing (per liter distilled water): 4.0 g peptone, 10.0 g glucose, 2.0 g Na_2_SO_4_, 1.0 g MgSO_4_·7H_2_O, 20.0 g NaCl, 0.5 g Mohr’s salt (FeSO_4_·(NH_4_)_2_SO_4_·6H_2_O), 0.1 g Na_2_S·9H_2_O, gas phase–argon, pH 7.2 [29]. The number of sulfate-reducing prokaryotes was determined by the formation of sulfide in the medium containing (per liter distilled water): 0.2 g KH_2_PO_4_, 0.25 g NH_4_Cl, 20.0 g NaCl, 0.5 g KCl, 3.0 g MgCl_2_·6H_2_O, 0.15 g CaCl_2_·2H_2_O, 4.0 g Na_2_SO_4_, 4.0 g Na lactate, 0.5 g yeast extract, 0.2 g Na_2_S·9H_2_O, NaHCO_3_ up to pH 7.0, gas phase—argon [30]. Methanogenic archaea were enumerated by the formation of methane in the gas phase on the medium containing (per liter distilled water): 0.2 g KH_2_PO_4_, 0.25 g NH_4_Cl, 20.0 g NaCl, 0.5 g KCl, 1.2 g MgCl_2_·6H_2_O, 0.15 g CaCl_2_·2H_2_O, 2.5 g Na acetate, 2 mL methanol, 0.5 g yeast extract, 2.5 g NaHCO_3_ [30], 1 mg rezazurin, 0.5 g Na_2_S·9H_2_O, pH 7.0–7.2. A mixture of H_2_/CO_2_ (4:1, *v*/*v*) was used as the gas phase. 1 mL·L^−1^ trace elements was added to each medium [31]. The cultures were incubated in stationary conditions in the dark for 20 days at 10 °C that was close to the average summer temperature in the Murmansk region.

#### 2.4.3. Isolation and Cultivation of Microorganisms

Pure bacterial cultures were isolated by inoculation of the bacteria from liquid media to solid media of the same composition containing 20 g·L^−1^ agar. The range and optimum salinity for the growth of microorganisms were determined using liquid PC medium with different NaCl content. Growth temperature limits were determined on PC medium with the optimal NaCl content for each strain. The cultures were incubated in stationary conditions for 7 days. In subsequent experiments, the optimal salinity and temperature conditions were used for each strain. The growth on hydrocarbon substrates was determined on MM mineral medium supplemented with 0.2% *v*/*v* of crude oil, diesel fuel or motor oil. The cultures were incubated at 10 °C for 30 days.

All cultures were studied using an Axio Imager.D1 epifluorescence microscope (Carl Zeiss, Oberkochen, Germany). Biofilm growth of oil-oxidizing bacteria in the sand samples was examined under a TM3000 scanning electron microscope (Hitachi, Tokyo, Japan).

### 2.5. Model Experiments on Oil Degradation in Sand by Pure Cultures

The most active oil-oxidizing bacteria were used in model experiments to purify sand from oil pollution. Calcined quartz sand (260 g) was introduced into a sterile plastic container, which was moistened to full moisture capacity with a mineral medium MM containing 20 g·L^−1^ NaCl. Then, approximately 0.3% (*w*/*w*) of sterile oil was added. Freshly grown cultures were inoculated in such way that the final number of bacteria was 10^7^ cells·g^−1^ of sand, and then the sand was thoroughly mixed. The cell number in the initial suspension was determined by MacFarland turbidity standards (bioMérieux, Marcy, l’Etoile, France). Sand inoculated with the culture without oil and uninoculated sand were used as controls. All experiments were carried out twice. Sand was incubated at 9 °C, and the moistening and loosening of sand was carried out once every 2 weeks. After 0, 30, and 60 days of cultivation, changes in the number of cells, the formation of biofilms, the formation of lower fatty acids (LFA) and alcohols, changes in the composition of the aliphatic fraction of oil, and the loss of oil were monitored. The biodegradation of oil was controlled by the weight method.

### 2.6. Analytical Methods

The increase in biomass was estimated spectrophotometrically by the change in the turbidity of the liquid medium at 600 nm. Methane, hydrogen, and CO_2_ in the headspace were determined by gas chromatography. Sulfide was determined colorimetrically with *p*-phenylenediamine by the method described by Trüper and Schlegel [32]. Volatile fatty acids and lower alcohols were analyzed using a GC-2010 Plus gas chromatograph (Shimadzu, Kyoto, Japan). The value of the surface tension at the culture liquid–air interface and the interfacial tension at the culture liquid–hexadecane interface was determined by the ring separation method using a Tensiomat 21 semi-automatic surface tensiometer (Cole-Parmer, Vernon Hills, IL, USA). Measurements were carried out at 20 °C. The emulsification index E_24_ was calculated 24 h after adding 1 mL of hexadecane to 1 mL of the culture liquid and intensive shaking for 3 min. The result was expressed as a percentage of the emulsion of the volume of the mixture. The control was a sterile medium.

The growth in oil was determined by the change in the content of *n*-alkane and *iso*-alkanes in the aliphatic fraction of degraded oil compared to the control (%) by gas–liquid chromatography using Kristall 5000.1 chromatograph (Khromatek, Yoshkar-Ola, Russia) with a flame ionization detector as described previously [33]. The chromatographic data were analyzed using the total height of phytane and pristane peaks (*iso*-C_19_ + *iso*-C_20_) for internal normalization.

### 2.7. Nucleotide Sequence Accession Number

Nucleotide sequences of the 16S rRNA gene of pure cultures were deposited into GenBank under accession nos: MW853692, MW853765, MW853771, MW853791, MW853833, MW854008, MW854024, MW854025, MW854029, MZ620649, MZ620650, MZ620656, MZ620679, MZ620680, MZ620683, MZ620702, MZ620714, MZ636810, and OM273845. The 16S rRNA gene fragment sequences of microbial communities were deposited to NCBI, project PRJNA738906.

## 3. Results and Discussion

### 3.1. Physicochemical Characteristics of Seawater, Littoral Ground and Coastal Soil Samples

Eight samples (two samples of sea water, four samples of littoral soil, and two samples of coastal soil) collected in the Murmansk region were studied by chemical methods. Seawater samples of the Kola Bay contained 11–14 g·L^−1^ NaCl and had a pH of 7.3–7.6; littoral and coastal soil samples contained 27–30 g·L^−1^ NaCl, pH 7.8–8.2. Ethanol (0–25 mg·L^−1^), acetate (18–121 mg·L^−1^), and C_3_–C_5_ lower fatty acids (LFA) were found in the studied samples (6–178 mg·L^−1^) (Table S1). In a sample of seawater 1_M21, taken from the Kola Bay in the area of the settlement Belokamenka, the highest levels of acetate and C_3_–C_5_ of LFA were noted (121 and 178 mg·L^−1^, respectively). Acetate at a concentration of 41–56 mg·L^−1^ was also present in soil samples of the littoral zone and coastal soil from the settlement of Belokamenka. In the samples of littoral soil taken near the settlements of Kola and Pechenga, the concentration of C_2_–C_5_ LFA did not exceed 39 mg·L^−1^.

### 3.2. Phylogenetic Diversity of Microbial Communities

**Figure 1:**
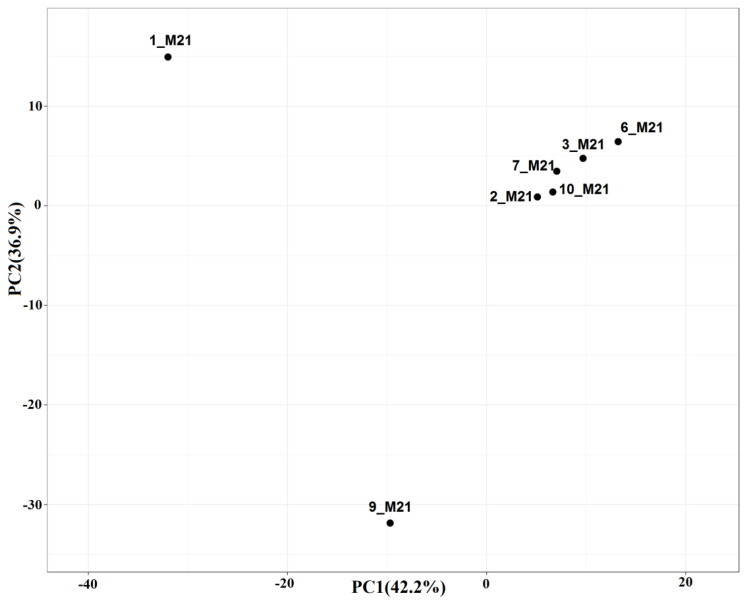
Comparison of the composition of microbial communities from seawater, littoral sandy mud, and coastal soil samples by principal component analysis (PCA) based on the relative abundance of operational taxonomic units (OTUs) derived from clustering the 16S rRNA genes (≥97% similarity) of prokaryotes. Library designations: prokaryotic communities in samples of seawater (1_M21 and 2_M21), littoral soil (3_M21, 6_M21, and 10_M21), and coastal soil (7_M21 and 9_M21) collected at the Murmansk region.

The composition of microbial communities of two seawater samples (1_M21, 2_M21) and five littoral and coastal soil samples (3_M21, 6_M21, 10_M21, 7_M21, 9_M21) was analyzed by high-throughput sequencing of the V4 region of the 16S rRNA gene. An attempt to isolate DNA from a coastal soil sample 11_M21 was unsuccessful, although microorganisms were detected in this sample by microbiological methods. The Good’s coverage index of the diversity of bacterial and archaeal phenotypes varied from 91 to 99%, which indicates the completeness of the libraries obtained (Table S2). Comparison of microbial diversity by principal component analysis (PCA) revealed that microbial communities fell into three groups (Figure 1). Communities of 1_M21 seawater from the area of the settlement of Belokamenka and coastal soil 9_M21 from the area of the settlement Roslyakovo represented two separate groups, whereas microbial communities of the remaining five out of seven samples formed the third group.

**Figure 2:**
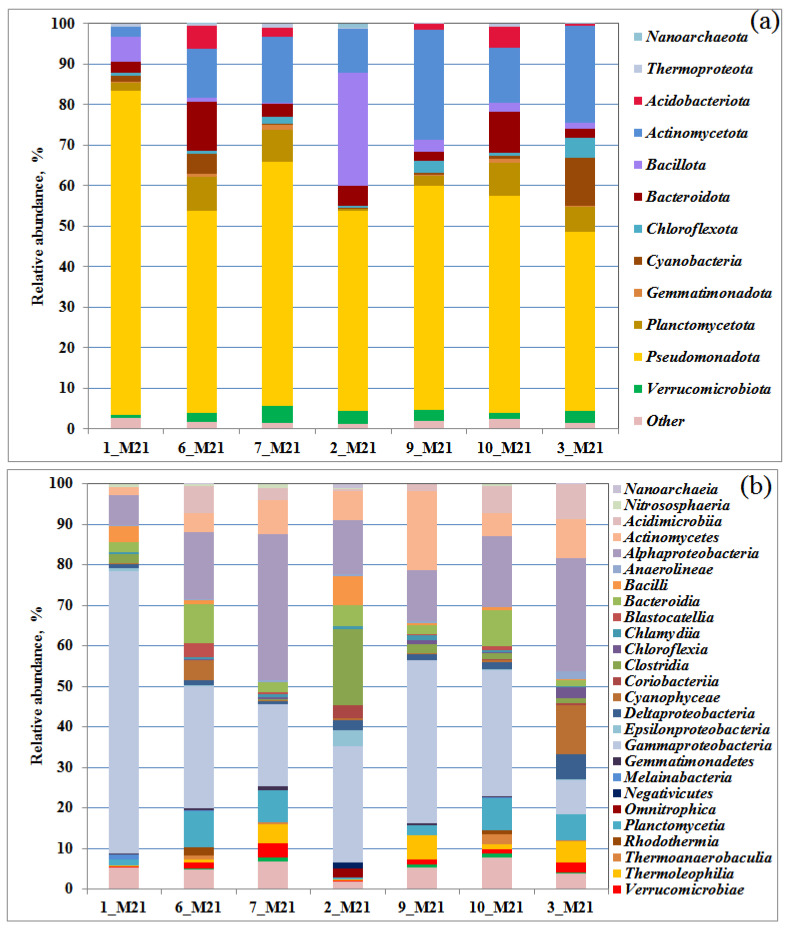
Relative proportion of bacterial 16S rRNA gene sequences presented at the phylum (**a**) and class level (**b**) in the libraries from seawater, littoral, and coastal soil samples. Bacterial taxa constituting > 1% in at least one library are listed.

**Figure 3:**
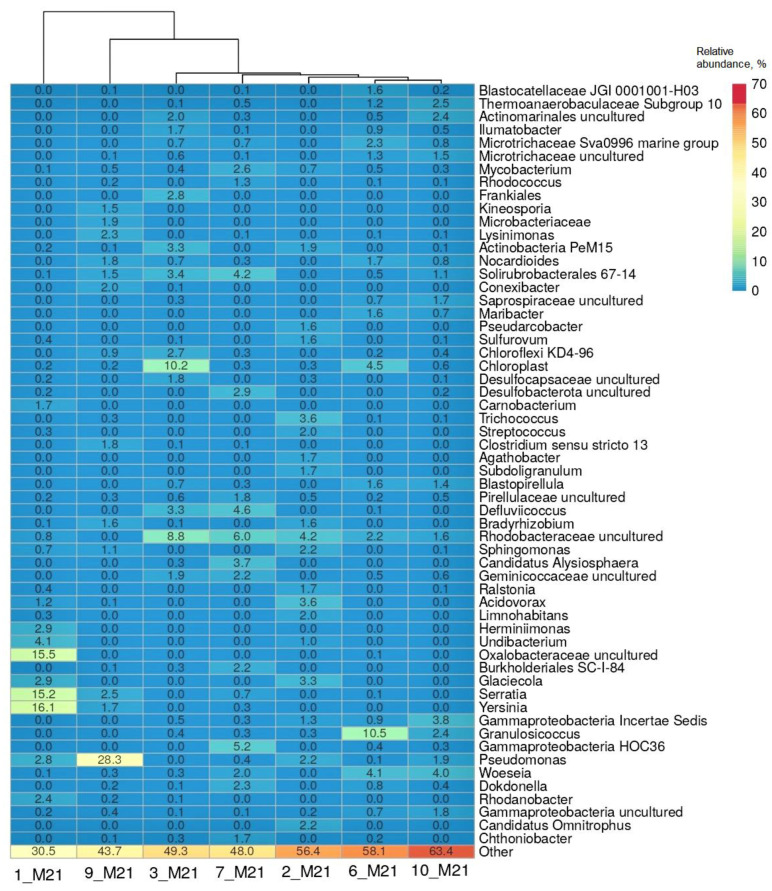
Heatmap of distribution of the genera with the highest relative abundance in the libraries of the 16S rRNA gene sequences from seawater, littoral, and coastal soil samples. Representation of the genus was calculated as sequence proportions divided by total sequence count in each library. Columns are clustered using correlation distance and average linkage.

**Figure 4:**
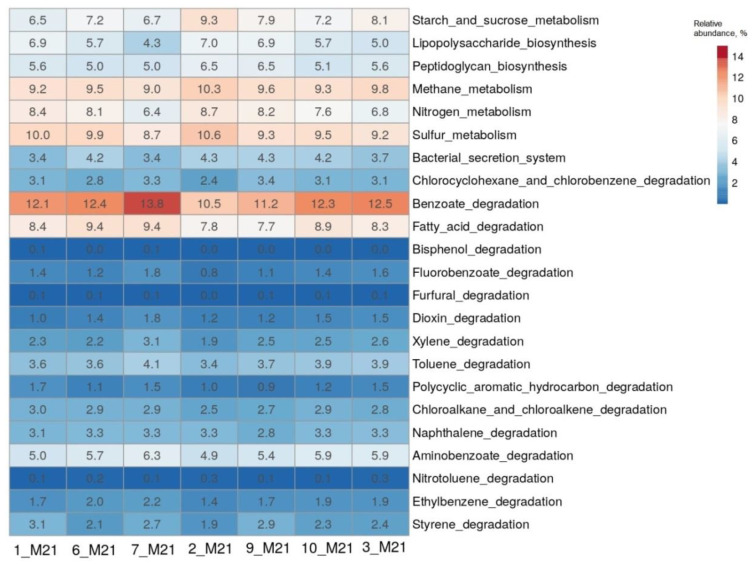
The heatmap showing the predicted functional profiles of studied microbial communities based on the KEGG Database.

The dominant microorganisms in the samples of seawater 1_M21 and coastal soil 9_M21 were different, which was confirmed by two separate groups they formed in the PCA figure, as well as by 16S rRNA gene sequencing and functional analysis (Figure 2, Figure 3 and Figure 4). The third group included samples of seawater, littoral (sandy mud) and coastal soils. The similarity of microbial communities in these samples can be explained by the mixing of seawater communities with communities of the littoral and coastal soils observed after the storm, as well as by stochastic causes. The reasons for this grouping can be determined by analyzing a large number of samples and a detailed analysis of the physicochemical conditions and the level of contamination.

The communities 1_M21 (seawater) and 9_M21 (coastal soil) contained 145 and 1124 OTUs, respectively. Littoral sandy mud communities 6_M21 and 10_M21 consisted of 1305 and 6257 OTUs, respectively, which were supported by highest CHAO indices and Shannon diversity indices (Table S2). Additional analysis of representative communities performed using the Venn diagram showed higher diversity of sandy mud communities located in the transition zone between seawater and coastal soil (Figure S2).

The quantitative distribution of the obtained 16S rRNA gene fragments in the libraries at the domain level is shown on Figure S3. Representatives of the bacteria domain predominated in all the samples studied; the share of archaea did not exceed 2% (of the total number of sequences in the libraries). The identified archaeal sequences belonged to the phyla Thermoproteota, Nanoarchaeota, Diapherotrites, *Candidatus* Woesearchaeota, and *Candidatus* Bathyarchaeota. In the samples of seawater and flooded littoral soil, ammonium-oxidizing archaea of the genera *Nitrosopumilus*, Ca. Nitrosotenuis, *Nitrosarchaeum*, and the *Nitrososphaeraceae* family were detected. Aerobic archaea of the genus *Nitrosopumilus* have also been previously identified in Arctic seas [34,35]. In the studied communities, Bacteria belonged to 12 main phyla (Figure 2a) and 27 classes (containing more than 1% of sequences in at least one library, Table S3). The bacteria of the phylum Pseudomonadota (=Proteobacteria, 44.0–79.9%), dominating in microbial communities, belonged to the classes Gammaproteobacteria (from 8.2 to 69.6%) and Alphaproteobacteria (from 7.4 to 36.1% of sequences in libraries) (Figure 2b).

The content of Gammaproteobacteria was the highest (69.6%) in a sample of seawater 1_M21 taken in the area of the village of Belokamenka, which is also characterized by a maximum content of C_2_–C_5_ lower fatty acids (LFA). The predominance of Gammaproteobacteria in the polar microbial communities was noted earlier [36,37]. The increase in the number of Pseudomonadota (=Proteobacteria), especially Gammaproteobacteria, has been considered a consequence of hydrocarbon pollution [20]. Alphaproteobacteria were most represented in soil samples 7_M21 and 3_M21, reaching 36.1 and 28.0% of the bacterial compositions, respectively. Representatives of the class Actinomycetes (phylum Actinomycetota), among which there are also hydrocarbon-oxidizing bacteria, were detected in all samples (from 1.9% to 19.6% of the libraries from samples 1_M21 and 9_M21, respectively).

The heatmap (Figure 3) shows a list of bacterial genera that comprised more than 1% of the sequences in at least one of the studied microbial communities. It should be noted that hydrocarbon-oxidizing bacteria are found in a number of listed genera, including *Acidovorax*, *Actinobacter*, *Bradyrhizobium*, *Burkholderia*, *Dokdonella*, *Granulosicoccus*, *Herminiimonas*, *Maribacter*, *Mycobacterium*, *Nocardioides*, *Pseudomonas*, *Ralstonia*, *Rhodanobacter*, *Serratia*, *Sphingomonas*, and *Streptococcus* [20].

The microbial community of the most polluted seawater sample 1_M21 was characterized by the dominance of a small number of taxa, namely, bacteria of the genera *Yersinia*, *Serratia* and uncultivated *Oxalobacteraceae* (accounting for 46.8% of the 16S rRNA gene sequences in the library); in the coastal soil sample 9_M21, the proportion of bacteria of the genus *Pseudomonas* reached 28.3%. Bacteria of the genus *Serratia* and the intestinal group of the genus *Yersinia* were among the dominant groups in the seawater sample 1_M21 (15.2% and 16.1%, respectively). The composition of microorganisms in the remaining samples was more aligned and included uncultivated bacteria belonging to the families *Rhodobacteraceae* and *Microtrichaceae* and classes Gammaproteobacteria and Actinomycetes, as well as organotrophic bacteria of the genera *Pseudomonas*, *Granulosicoccus*, *Woeseia*, *Mycobacterium*, *Defluviicoccus*, *Dokdonella* and others (Figure 3). Uncultivated sulfate-reducing bacteria of the *Desulfocapsaceae* family and Deltaproteobacteria class were found in the samples of littoral soil (3_M21 and 10_M21) and coastal soil 7_M21.

### 3.3. Potential Functional Characteristics of the Studied Microbial Communities

The results of the determination of the composition of microbial communities based on the analysis of 16S rRNA genes were further analyzed using the iVikodak program [26]. The potential functional characteristics of the studied bacterial communities were predicted using the “Global Mapper” module of the iVikodak database. From the heatmap of the comparison of functional profiles, it can be seen that microorganisms made a significant contribution to the metabolism of sulfur, nitrogen, methane, starch and sucrose, and the degradation of fatty acids (Figure 4). The enzymes of degradation of benzoate, which is the central metabolite of degradation of various aromatic compounds, were most represented in the microorganisms of all the samples studied. The contribution of each community to these metabolic pathways ranged from 6.5 to 13.8%. Predicted enzyme profiles for the metabolism of the above compounds and the key microorganisms involved in these pathways are presented in Figures S4–S9 and in Tables S4–S9, respectively.

The predicted enzyme profiles for the “Nitrogen metabolism” pathway (KEGG Orthology (KO) pathway KO00910) in microorganisms of the seawater sample 1_M21 and littoral soil sample 3_M21 were similar (Figure S4) and included enzymes of the pathway of assimilatory nitrate reduction to ammonium, i.e., *NarB* (assimilatory ferredoxin-nitrate reductase (EC 1.7.7.2)) and *NR* (nitrate reductase (NAD(P)H) (EC:1.7.1.1 1.7.1.2 1.7.1.3)), *NIT-6* (nitrite reductase (NAD(P)H) (EC:1.7.1.4)), and *NirA* (ferredoxin-nitrite reductase (EC:1.7.7.1)). Predicted enzymes responsible for nitrite reduction to ammonium in the dissimilatory nitrate reduction pathway included *NirB* (nitrite reductase (NADH) large subunit (EC:1.7.1.15)), and *NrfA* (nitrite reductase (cytochrome c-552) (EC:1.7.2.2)). Predicted enzymes of incomplete denitrification pathway participating in sequential reduction of nitrite via nitric oxide and nitrous oxide to dinitrogen (Figure S4) included *NirK* (nitrite reductase (NO-forming) (EC:1.7.2.1)), *NirS* (nitrite reductase (NO-forming) / hydroxylamine reductase (EC:1.7.2.1 1.7.99.1)), *NorB* (nitric oxide reductase subunit B (EC:1.7.2.5)), and *NosZ* (nitrous-oxide reductase (EC:1.7.2.4)).

Using the ”Global Mapper" module of the iVikodak program, the contribution of bacteria of various taxa to the implementation of the “Nitrogen metabolism” pathway was evaluated. The contribution to nitrogen metabolism exceeding 10% was made by various bacteria of the genera *Pseudomonas*, *Serratia*, *Blastopirellula*, *Yersinia*, *Nocardioides*, *Mycobacterium*, *Trichococcus*, *Rhodanobacter*, *Acidovorax*, *Glaciecola*, and *Maribacter* and uncultured members of *Solirubrobacterales*, *Oxalobacteraceae*, and *Rhodobacteraceae* (Table S4). Bacteria of the genera *Pseudomonas* and *Serratia* predominated in the seawater sample 1_M21. A number of bacteria of the genus *Pseudomonas* are able to fix dinitrogen; they are also known as efficient denitrifiers [38]. Bacteria of the genus *Serratia* are able to effectively remove ammonium from polluted waters by participating in the nitrogen cycle [39]. In members of the genus *Serratia*, nitrogen fixation has recently been demonstrated in conjunction with As(III) oxidation, a new process identified in mine tailings [40].

The enzymes of the pathway “Sulfur metabolism” (KO00920) were most represented in microorganisms of seawater samples 1_M21 and 2_M21 (Figure 4). The bacteria possessed mainly the enzymes of assimilatory sulfate reduction, catalyzing the reduction of sulfate to adenylyl sulfate, then to sulfite and sulfide. Nevertheless, the enzyme 3′-phosphoadenosine 5′-phosphosulfate synthase (EC:2.7.7.4), catalyzing the reduction of sulfate to adenylyl sulfate, was also predicted in microorganisms of seawater 2_M21 and littoral soil 3_M21 samples, as well as the enzymes of subsequent reduction of adenylyl sulfate to sulfite and further to sulfide in the path of dissimilatory sulfate reduction, probably belonging to sulfate-reducing bacteria of the class Deltaproteobacteria (Figure S5; Table S5).

Communities of seawater 1_M21 and littoral soil 3_M21 contained bacteria possessing the key enzymes of the aerobic degradation pathway of benzoate (KO00362, “Benzoate degradation I”), benzoate-1,2-dioxygenase (EC: 1.14.12.10) and dihydroxycyclohexadiene dehydrogenase (EC: 1.3.1.25), which catabolize benzoate to catechol (Figure S6). Bacterial communities were predicted to have all catechol catabolism enzymes, both to succinyl-CoA and to pyruvate/acetyl-CoA. The bacteria of both communities also had the enzyme benzoate-CoA ligase (EC:6.2.1.25), catalyzing the conversion of benzoate to benzoyl-CoA—an intermediate of the biodegradation of many aromatic compounds. Several enzymes of the benzoate degradation pathway (“Benzoate degradation II”) via benzoyl-CoA, carried out mainly by anaerobic bacteria, have also been found. In the seawater community 1_M21, uncultivated Betaproteobacteria of the *Oxalobacteraceae* family and Gammaproteobacteria of the genera *Serratia*, *Yersinia*, and *Pseudomonas* were responsible for benzoate degradation, and in the littoral soil community 3_M21, bacteria of the genera *Nocardioides* (Actinomycetes class), *Blastopirellula* (Planctomycetia class), and cultivated (*Rhodanobacter*) and uncultivated Gammaproteobacteria (Table S6), participated in this process.

The analysis of the “Polycyclic aromatic hydrocarbon degradation" pathway (KO00624) showed that the studied microbial communities had almost no ability to degrade many PAHs (Figure S7). However, bacteria of the seawater sample 1_M21 and of coastal soil sample 9_M21 possessed a complete set of enzymes for terephthalate catabolism to 3-carboxy-*cis*,*cis*-muconate or 4-carboxy-2-hydroxymuconate semialdehyde, which are further transformed along the benzoate degradation pathway. Terephthalate is an intermediate product of degradation of polyethylene terephthalate (PET), widely used in Russia for the production of food films and plastic bottles, in shipbuilding for construction of bio-resistant hull parts and other industries. PET in the composition of microplastics is one of the main pollutants of marine ecosystems and causes great environmental damage [41,42]. Polyethylene terephthalate belongs to the group of aliphatic–aromatic polyesters; it is chemically and thermally stable. Although a number of bacteria capable of destroying polyester materials are known, genes and key enzymes of the PET degradation pathway have been reliably studied only for the bacterium *Ideonella sakaiensis* [43,44,45]. Potential terephthalate degraders in the microbial communities of the 1_M21 seawater sample and of the 9_M21 coastal soil sample were members of the genus *Pseudomonas* (Table S7), for which terephthalate degradation enzymes were studied [46].

Enzymes of *n*-alkanes degradation are considered in the “Degradation of fatty acids” pathway (KO00071). The main enzyme of *n*-alkanes degradation, alkane-1-monooxygenase, was present in bacteria from all the samples studied (Figure S8). The greatest predicted contribution to the degradation of fatty acids was made by bacteria of the genera *Pseudomonas*, *Mycobacterium*, *Nocardioides*, *Acidovorax*, *Sphingomonas*, *Serratia*, *Blastopirellula*, and *Maribacter* and of the families *Rhodobacteraceae* and *Oxalobacteraceae* (Table S8), among which bacteria that degrade alkanes are known [20].

The enzymes of the metabolism of methane and single-carbon compounds (KO00680, Figure S9), as well as the key microorganisms involved in these processes (Table S9) were predicted for the studied bacterial communities. Due to the oxidized habitat conditions, microbial communities mainly carried out the processes of aerobic oxidation of methane and single-carbon compounds. No methanogens were detected in the studied samples. However, heterotrophic bacteria of the genus *Pseudomonas* can participate in the aerobic formation of methane due to the demethylation of methylphosphonic acid polysaccharide esters that are present in organic matter [47]. The genes responsible for C1-conversion and participation in the methane cycle were previously found in the genome of bacteria of the genus *Blastopirellula*, but their functions are still not elucidated [48].

Bacteria of the genus *Pseudomonas*, among which both mesophilic and psychrophilic oil degraders have been described, were identified in almost all the communities studied. Members of the genus *Pseudomonas* are resistant to heavy metals, nitrate, various organic pollutants, and crude oil, which causes their widespread distribution in polluted habitats. Psychrophilic strains of *Pseudomonas* spp. were identified in Arctic ecosystems contaminated with oil and petroleum products [49,50]. *Pseudomonas* bacteria are a model object for studying the biodegradation of a wide range of organic pollutants, including crude oil, polycyclic aromatic compounds, benzoate, catechol, toluene, etc. [50]. Among the representatives of this genus, there are known bacteria that fix molecular nitrogen, reduce oxidized nitrogen compounds to ammonium or molecular nitrogen, psychrotolerant phosphate-solubilizing bacteria, as well as reducing oxidized forms of metals, metalloids, and radionuclides [40,51].

Chemo-organoheterotrophic facultatively anaerobic bacteria of the genus *Woeseia* were found in six of the seven communities studied, where they accounted for no more than 4.1%. Currently, the genus *Woeseia* is represented by a single species *Woeseia oceani*, first isolated from coastal sediments of China [52]. These bacteria grow at 8 °C, although the optimal temperature is 28–35 °C. *Woeseia* bacteria were also found in the microbial community of bottom sediments in the Gulf of Mexico, and the genes determining the degradation of naphthalene, polycyclic aromatic hydrocarbons (PAH), and alkanes were identified in their genome [53]. Bacteria of the genus *Granulosicoccus* were found in five samples studied; they were most represented (10.5%) in the 6_M21 sample of littoral soil. This genus includes psychrophilic aerobic organotrophic bacteria, which have been previously found in microbial communities utilizing hydrocarbons [54,55].

Bacteria of the genus *Serratia* are known to grow at low temperature on crude oil with the formation of biosurfactants [56]. Psychrotolerant strains of *Yersinia* sp., isolated earlier from a lake on Svalbard in the Arctic zone, were capable of biosorption of arsenic [57]. Bacteria of the genera *Acidovorax* and *Glaciecola* were also present in both seawater samples 1_M21 and 2_M21. Some hydrocarbon-oxidizing bacteria of the genus *Acidovorax* grow on PAHs, for example, on phenanthrene [58]. *Glaciecola*-*Paraglaciecola* degrade sugar-containing compounds and polysaccharides of algae; they are often found in low-temperature Arctic and Antarctic marine habitats [59]. Psychrophilic bacteria of the genus *Glaciecola* and potential oil-oxidizing bacteria of the genera *Sphingomonas* and *Dokdonella* were also found in the bacterial community of the Arctic surface waters of the zone between the Bering Strait and the Chukchi borderland [60].

Among the bacteria of the genus *Nocardioides*, there are also psychrophilic representatives isolated from cold habitats; for the species *Nocardioides oleivorans*, growth on oil was shown [61,62]. Members of the genus *Mycobacterium* are also known degraders of various aromatic hydrocarbons, which was confirmed by discovery of the relevant genes [12]. Despite the fact that bacteria of the genus *Blastopirellula* are found in oil-contaminated habitats, we have not found evidence that these microorganisms are involved in the degradation of hydrocarbons [63]. Uncultivated representatives of the *Rhodobacteraceae* family were identified in most of the samples studied. This family includes bacteria inhabiting marine ecosystems, for example, bacteria of the genus *Roseobacter*, which have been repeatedly isolated from polar microbial communities and for which the ability to use aromatic hydrocarbons and alkanes has been shown [64].

Thus, the studied microbial communities of seawater and soils included bacteria potentially capable of degradation of a wide range of crude oil components.

### 3.4. Cultivable Microorganisms from Samples of Water, Littoral and Coastal Soils

Aerobic organotrophs, including hydrocarbon-oxidizing bacteria, as well as bacteria with a fermentative type of metabolism, were detected in all samples inoculated in nutrient media (Figure S10). The maximum number of aerobic organotrophic and hydrocarbon-oxidizing bacteria reached 10^7^ cells·g^−1^ in a sample of coastal soil 9_M21. Hydrocarbon-oxidizing bacteria were present in both seawater samples (10^4^ cells·mL^−1^). The number of fermentative bacteria ranged from 10^3^ to 10^6^ cells·mL^−1^ (g^−1^). Methanogenic archaea were not detected in the samples studied. Single cells of sulfate-reducing bacteria were found only in a sample of littoral soil 3_M21. The absence of strict anaerobes was probably due to the shallow sampling depth and the oxidized environment.

### 3.5. Isolation and Identification of Pure Cultures of Aerobic Bacteria

Primary enrichment cultures obtained on the media for aerobic organotrophic and hydrocarbon-oxidizing bacteria were used for the isolation of pure cultures. The 16S rRNA gene sequences of the 19 isolated bacterial strains had more than 99.6% similarity with the genes of the type strains of the validly described taxa. These strains were assigned to 15 known species of seven genera belonging to the orders Pseudomonadales (genus *Pseudomonas*), Aeromonadales (*Aeromonas*, *Oceanisphaera*), Alteromonadales (*Shewanella*), and Enterobacterales (*Serratia*) of Gammaproteobacteria class, as well as to actinobacteria of the orders Micrococcales (*Paeniglutamicibacter*) and Corynebacteriales (*Rhodococcus*) (Table S10). Nine isolated strains belonged to the genus *Pseudomonas*, whose representatives were identified by molecular methods in most of the samples studied.

Isolates were phylogenetically close to *Pseudomonas guineae* (strain M3-10) and *Pseudomonas leptonychotis* (strain M11-3), psychrotolerant bacteria from Antarctic habitats, *Pseudomonas baetica* (strains M9-22 and M7-26), a fish pathogen, and *Pseudomonas protegens* (strain M7-27) previously isolated from wastewater of a pesticide processing plant [10,65,66,67]. Strain M11-25 was assigned to the species *Pseudomonas kielensis* [68], capable of growing within the temperature range from 4 to 30 °C. Bacteria of the genus *Rhodococcus* were detected by molecular method only in the sample of coastal soil 7_M21. However, pure cultures of *Rhodococcus erythropolis* M7-8 and M2-15 [69] and *Rhodococcus fascians* M6-11 and M6-12 were isolated from seawater, littoral, and coastal soil (2_M21, 6_M21 and 7_M21) sampled near the settlements of Belokamenka and Roslyakovo.

The use of high-throughput sequencing for the study of polar microbial communities makes it possible to obtain more comprehensive information on the biodiversity of microorganisms compared to cultural methods; however, some of the isolated microorganisms are sometimes not detected by molecular methods [70]. We also failed to find bacteria of the genera *Aeromonas*, *Paeniglutamicibacter*, *Oceanisphaera*, and *Shewanella* by the 16S rRNA gene-based approach, although these bacteria were later isolated into pure cultures. This may be caused by the low representation of these bacteria in the communities and PCR preferences.

Strain M10-21 was phylogenetically close to the bacterium *Paeniglutamicibacter psychrophenolicus* (formerly *Arthrobacter psychrophenolicus*) growing on phenol at 1–25 °C and isolated from an ice cave [71]. Strains M6-13 and M6-14 were assigned to the species *Oceanisphaera marina*, which are common inhabitants of seawater [72]; psychrophilic representatives of this genus have also been isolated from Arctic soils [73]. Strain M3-1 was close to the facultatively anaerobic mesophilic bacterium *Aeromonas salmonicida* subsp. *pectinolytic* isolated from a polluted river in Argentina [74]. The genus *Aeromonas* includes both mesophilic and psychrophilic bacteria living in river or sea water, some subspecies belong to fish pathogens [74,75]. *Aeromonas salmonicida* grows on hydrocarbons (gasoline) and produces biosurfactants [75].

Strain M3-18 was assigned to the species *Shewanella livingstonensis*. Hydrocarbonoclastic bacteria of the genus *Shewanella* were previously isolated from Antarctic seawater [76], found as part of a microbial community growing on crude oil in conditions simulating seawater of the Arctic region [77]. Oil-degrading *Shewanella* cultures were also isolated from oil reservoirs [78,79] and seawater of the Persian Gulf [80,81]. The formation of glycolipid biosurfactants by a marine isolate *Shewanella algae* B12 has been shown [82]. Members of the genus *Shewanella* are able to utilize a range of organic substrates, to reduce iron and a number of other metals, metalloids, and radionuclides [83,84]; their presence in a sample of littoral soil 3_M21 may be a consequence of metal contamination of coastal areas and seawater, noted earlier (http://mpr.gov-murman.ru (accessed on 21 June 2022)). Thus, bacteria of the genera *Aeromonas*, *Oceanisphaera*, *Paeniglutamicibacter*, *Pseudomonas*, *Rhodococcus*, *Serratia*, and *Shewanella* have previously been repeatedly detected as part of psychrotolerant and/or oil-degrading microbial communities.

### 3.6. Degradation of Crude Oil and Petroleum Products by Isolated Strains

**Figure 5:**
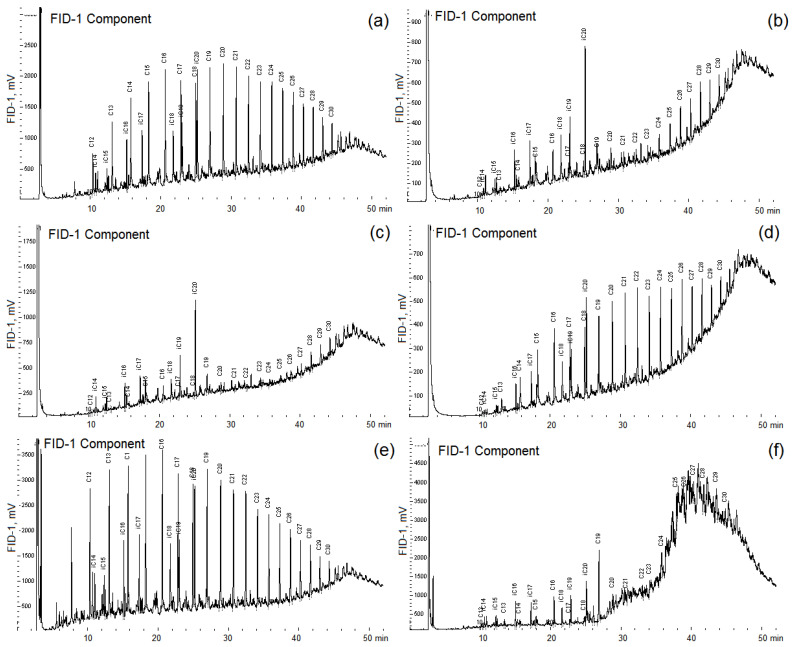
Chromatograms of *n*-alkanes of crude oil (control, (**a**)) and of oil degraded by *Pseudomonas brenneri* M6-6 (**b**), *Rhodococcus erythropolis* M7-8 (**c**), *Serratia myotis* M7-5 (**d**), *Pseudomonas kielensis* M11-25 (**e**), and by *Rhodococcus erythropolis* M2-15 (**f**). Strains were incubated in a medium with 0.2% (*v*/*v*) crude oil for 30 days at 10 °C.

Many bacteria of the genera *Pseudomonas*, *Rhodococcus*, and *Paeniglutamicibacter* oxidize oil and are often components of the biological preparations for removing of oil pollution. All isolated strains were tested for the ability to grow utilizing crude oil and petroleum products in a liquid medium. Strains were incubated in the liquid medium with 0.2% (*v*/*v*) crude oil for 30 days at 10 °C. The concentration of oil added to the medium corresponded to a low level of pollution but made it possible to monitor the utilization of various alkanes of oil by gas chromatography, which would be difficult with a higher concentration of oil in the medium. The most significant utilization of crude oil *n*-alkanes compared with the control was observed for strains *Aeromonas salmonicida* M3-1, *Rhodococcus erythropolis* M7-8 and M2-15, *Pseudomonas brenneri* M6-6, *Pseudomonas leptonychotis* M11-3 (Figure 5, Figure S11). A decrease in the proportion of total *n*-alkanes in oil degraded by the isolates of hydrocarbon-oxidizing bacteria relative to that in the crude oil in the control sample confirmed utilization of these oil components by the studied strains (Figure S12). The strain *R. erythropolis* M7-8 used mainly unbranched C_19_–C_30_ alkanes, while the biodegradation of alkanes with a shorter chain length was also significant (Figure S11). Growth of the studied strains on the medium- and long-chain *n*-alkanes was characterized. When growing on oil, strains *P. kielensis* M11-25, *P. psychrophenolicus* M10-21, *S. livingstonensis* M3-18, and *O. marina* M6-13 weakly used *n*-alkanes with a chain length of more than C_18_ and did not grow on short-chain *n*-alkanes.

The *Serratia myotis* strain M7-5, on the contrary, used only C_12_–C_20_ *n*-alkanes and did not use *n*-alkanes with a longer carbon chain (Figure 5). The growth of pure cultures on crude oil was accompanied by a change in the rheological characteristics of the culture liquid, which indicated the formation of biosurfactants. The high decrease in the surface and interfacial tension was shown for culture liquids of *A. salmonicida* M3-1 and *R. erythropolis* M7-8 strains (Table S11). Five strains efficiently degrading oil were studied in more detail (Table 1).

These strains grew at a low temperature of 5 °C and may be considered psychrotolerant bacteria. However, the maximum growth rate at temperatures below 20 °C, which is characteristic of psychrophilic bacteria, was observed in four strains, *A. salmonicida* M3-1, *P. brenneri* M6-6, *P. kielensis* M11-25, and *R. erythropolis* M7-8. Strain *R. erythropolis* M2-15 was a mesophilic bacterium capable of growth within the range of 5–37 °C, with an optimum at 30 °C. All five strains grew in the absence of NaCl in the medium, were tolerant to the presence of salt, the upper limit for growth varied from 6 to >7.5% NaCl (*w*/*v*) in the medium. These strains used diesel fuel, mineral, and motor oils at low temperatures.

**Figure 6:**
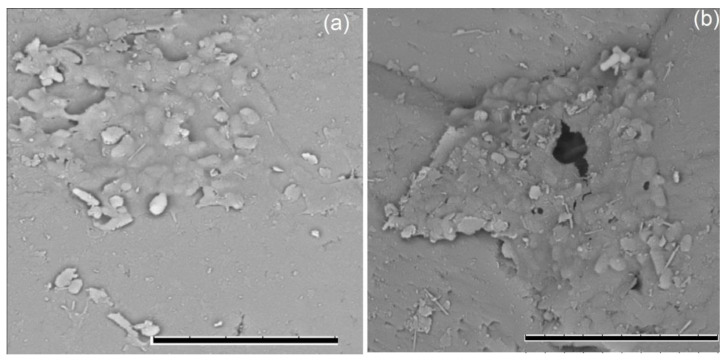
Formation of biofilms on sand with 0.3% (*w*/*w*) crude oil by strains of *Aeromonas salmonicida* M3-1 (**a**) and *Pseudomonas brenneri* M6-6 (**b**) after 30 days of incubation at 9 °C; metal-sprayed dry cells under a TM3000 scanning electron microscope (Hitachi, Tokyo, Japan) at 15 kV accelerating voltage. Bar, 10 µm.

The strains *R. erythropolis* M2-15 and *P. brenneri* M6-6 were used in model experiments to remove oil from sand. After 30 days of cultivation, the number of cells decreased from 10^7^ to 10^5^–10^6^ cells·g^−1^ in the variants with sand contaminated with oil and to 10^4^–10^6^ cells·g^−1^ for control sand without oil for both studied strains. On the 60th day, the number of cells in the sand with oil was gradually restored and amounted to 5 × 10^5^–10^7^, while in the variants without oil it varied within 10^4^–10^6^ cells·g^−1^. In the course of the experiment, the formation of biofilms on sand with oil was noted (Figure 6).

Growth of *P. brenneri* strain M6-6 was accompanied by the accumulation of acetone (48 mg·L^−1^), ethanol (95 mg·L^−1^), and acetate (9 mg·L^−1^) in the medium, production of which by strain M2-15 was at the detection limit. Bacterial growth was accompanied by changes in the composition of the aliphatic fraction of oil (Figure S11). After 60 days of cultivation, less than 50% of *n*-alkanes remained, and not only straight-chain, but also *iso*-alkanes were utilized. The loss of oil determined by the weight method in the control experiment was 24% (*w*/*w*), while it reached 36 and 38% (*w*/*w*) as a result of biodegradation by *P. brenneri* M6-6 and *R. erythropolis* M2-15, respectively. These levels of hydrocarbon degradation are in agreement with the literature data [85]. It is known that the combination of strains of various species increases the degree of oil biodegradation [3]. The investigation of hydrocarbon degradation by these strains in consortium is an essential part of the development of bioremediation biotechnologies for oil-contaminated soils. There is information that the bacteria found in the microbial communities by the 16S rRNA gene sequencing, as well as members of the genera isolated in pure cultures, are able to utilize not only *n*-alkanes, but also a number of other oil components, including polycyclic aromatic hydrocarbons, under aerobic or anaerobic conditions [86,87,88,89,90]. The data obtained indicate that the studied strains can be recommended for the application in biotechnologies for oil removal at low temperature. It is widely believed that bioaugmentation of oil-contaminated soils by hydrocarbon-oxidizing associations in combination with biostimulation (the introduction of mineral fertilizers) gives the same results of oil degradation as the use of biostimulation of autochthonous hydrocarbon-oxidizing soil microbiota by applying mineral fertilizers, and only slightly accelerates this process [8,91,92]. However, in some cases, hydrocarbons of different classes are extracted from sediments or soil contaminated with petroleum products, and the resulting solutions are treated ex situ in bioreactors with immobilized cells [93]. In such cases, effective oil degraders, including the isolated psychrophilic strains, may be used.

## 4. Conclusions

The development of natural resources of the Arctic in the northern regions of Russia and technogenic pollution with petroleum products can lead to environmental changes in marine and coastal ecosystems. In this work, the phylogenetic diversity of prokaryotes in the samples of Arctic seawater and coastal soil in the zone of anthropogenic impact in the Murmansk region and the potential functional capabilities of microorganisms in response to oil pollution was determined, and psychrophilic oil-oxidizing bacteria were isolated. By the 16S rRNA gene sequencing, members of approximately 20 genera of bacteria known for their ability to degrade hydrocarbons were found in the studied microbial communities. Among the dominant classes of bacteria, functionally diverse representatives of Gammaproteobacteria and Alphaproteobacteria were present in all types of samples—seawater, littoral (sandy muds) and coastal soils. Members of Actinomycetes, Acidimicrobiia, and Planctomycetia mainly predominated in the samples of sandy muds and coastal soils, characterized by lower pH and salinity in comparison to seawater. Cyanobacteria prevailed in the illuminated sandy muds flooded with seawater. Using the taxonomic abundance of OTUs and the KEGG database, the potential contribution of the studied bacterial communities to the nitrogen and sulfur cycles, degradation of benzoate, terephthalate, fatty acids, and alkanes was predicted. By cultural methods, bacterial strains of the genera *Pseudomonas*, *Serratia*, and *Rhodococcus*, as well as of the genera *Aeromonas*, *Oceanisphaera*, *Paeniglutamicibacter*, and *Shewanella*, which “escaped” from sequencing, were isolated. Among the 19 isolated strains, hydrocarbon-oxidizing bacteria were found, probably taking part in the process of self-purification of their habitats from hydrocarbons. Psychrophilic and psychrotolerant strains *Rhodococcus erythropolis* M2-15 and M7-8, as well as *Pseudomonas brenneri* M6-6, have a wide range of salinity for growth and are able to use *n*-alkanes of crude oil and diesel fuel to form biosurfactants, which makes them promising for application in bioremediation of Arctic habitats from hydrocarbon pollution at low temperature.

## Figures and Tables

**Table 1:** Physiological characteristics of isolated hydrocarbon-oxidizing bacteria.

Species, Strain	Temperature Range (Optimum), °C	NaCl Range (Optimum), % (*w*/*v*)	Utilization at 10 °C and 2% (*w*/*v*) NaCl		
			Diesel Fuel	Mineral Oil	Motor Oil
*Aeromonas salmonicida* M3-1	5–42 (15)	0–7.5 (0–1)	+ *	+	+
*Pseudomonas brenneri* M6-6	5–35 (10–15)	0–>7.5 (0.5)	+	+	+
*Pseudomonas kielensis* M11-25	5–35 (15)	0–6 (0–1)	+	+	+
*Rhodococcus erythropolis* M2-15	5–37 (30)	0–7.5 (2)	+	+	+
*Rhodococcus erythropolis* M7-8	5–37 (15–30)	0–>7.5 (0–1)	+	+	+

* +, Positive result.

## Data Availability

The libraries of 16S rRNA gene fragments of microbial communities were deposited in NCBI SRA, project no. PRJNA738906. The GenBank/EMBL/DDBJ accession numbers of the 16S rRNA gene sequence of pure cultures were deposited under accession nos: MW853692, MW853765, MW853771, MW853791, MW853833, MW854008, MW854024, MW854025, MW854029, MZ620649, MZ620650, MZ620656, MZ620679, MZ620680, MZ620683, MZ620702, MZ620714, MZ636810, and OM273845.

## Supplementary Materials

The following supporting information can be downloaded at: https://www.mdpi.com/article/10.3390/microorganisms10081490/s1, Table S1: The content of alcohols and lower fatty acids (mg·L^−1^ sea water or mg·g^−1^ soil) in the studied samples of seawater, littoral sandy mud, and coastal soil collected at the Murmansk region; Table S2: The number of the 16S rRNA gene sequences and operational taxonomic units (OTUs), coverage and diversity indices in libraries of bacterial communities of the seawater (1_M21 and 2_M21), littoral sandy mud (3_M21, 6_M21, and 10_M21), and coastal soil (7_M21 and 9_M21) samples; Table S3: Heatmap showing major bacterial classes proportions based on 16S rRNA gene amplicon sequencing with taxonomic classification using the SILVA database in samples of seawater, littoral sandy mud, and coastal soil; Table S4: Contribution of key bacteria to the metabolism of nitrogen compounds in the studied microbial communities (%); Table S5: Contribution of key bacteria to the metabolism of sulfur compounds in the studied microbial communities (%); Table S6: Contribution of key bacteria to benzoate metabolism in the studied microbial communities (%); Table S7: Contribution of key bacteria to the metabolism of polycyclic aromatic hydrocarbons in the studied microbial communities (%); Table S8: Contribution of key bacteria to the degradation of fatty acids in the studied microbial communities (%); Table S9: Contribution of key bacteria to methane metabolism in the studied microbial communities (%); Table S10: Taxonomic affiliation of pure bacterial cultures isolated from samples of seawater, littoral soil and coastal soil; Table S11: Surface tension (ST) and interfacial tension (IT) of the culture liquid of aerobic bacteria grown in a medium with crude oil at 10 °C for 30 days; Figure S1: Sampling sites at the Murmansk region; Figure S2: Venn diagram of the OTUs in the seawater (1_M21), littoral sandy mud (6_M21 and 10_M21) and coastal soil (9_M21) samples; Figure S3: Relative abundance of the 16S rRNA gene fragments of the bacteria and archaea in the libraries of prokaryotic communities in samples of seawater (1_M21 and 2_M21), of littoral soil (3_M21, 6_M21, and 10_M21), and of coastal soil (7_M21 and 9_M21) collected at the Murmansk region; Figure S4: Predicted enzyme profiles for “Nitrogen metabolism” pathway (KO00910) in microorganisms of the seawater 1_M21 (left) and littoral soil 3_M21 (right) samples; Figure S5: Predicted enzyme profiles for “Sulfur metabolism” pathway (KO00920) in microorganisms of the seawater 1_M21 (left) and littoral soil 3_M21 (right) samples; Figure S6: Predicted enzyme profiles for “Benzoate degradation” pathway (KO00362) in microorganisms of the seawater 1_M21 (upper) and littoral soil 3_M21 (lower) samples; Figure S7: Predicted enzyme profiles for “Polycyclic aromatic hydrocarbon degradation” pathway (KO00624) in microorganisms of the seawater 1_M21 (left) and coastal soil 9_M21 (right) samples; Figure S8: Predicted enzyme profiles for “Fatty acid degradation” pathway (KO00071) in microorganisms of the seawater 1_M21 (left) and coastal soil 9_M21 (right) samples; Figure S9: Predicted enzyme profile for “Methane metabolism” pathway (KO00680) in microorganisms of the seawater 1_M21 (upper) and littoral soil 3_M21 (lower) samples; Figure S10: Cells number of aerobic organothrophic bacteria (AOB), hydrocarbon-oxidizing bacteria (HOB), and fermentative bacteria (FB) in samples of seawater (W), littoral sandy mud (LS), and coastal soil (S) collected at the Murmansk region; Figure S11: Residual content of *n*-alkanes in the aliphatic fraction of oil degraded by strains *Aeromonas salmonicida* M3-1 (a,b), *Pseudomonas brenneri* M6-6 (c,d), and *Rhodococcus erythropolis* M2-15 (e,f) in a liquid medium with oil for 30 days (a,c,e) and on sand with oil at 10 °C for 60 days (b,d,f); Figure S12. The utilization of crude oil *n*-alkanes by pure cultures *Aeromonas salmonicida* M3-1, *Pseudomonas leptonychotis* M11-3, *Serratia myotis* M7-5, *Pseudomonas brenneri* M6-6, *Rhodococcus erythropolis* M7-8, *Pseudomonas silesiensis* M9-9, *Pseudomonas guineae* M3-10, *Oceanisphaera marina* M6-14, *Shewanella livingstonensis* M3-18, *Pseudomonas baetica* M9-22, *Pseudomonas kielensis* M11-25, and *Pseudomonas protegens* M7-27.
