# Supplementary material for: Microbial Communities of Seawater and Coastal Soil of Russian Arctic Region and Their Potential for Bioremediation from Hydrocarbon Pollutants
Source: Microorganisms. 2022 Jul 24;10(8):1490. doi: 10.3390/microorganisms10081490 (PMC9332119; doi:10.3390/microorganisms10081490)
Supplement: Supplementary file 1 [file microorganisms-10-01490-s001.zip › microorganisms-1804833-supplementary.pdf]

microorganisms

MDPI

Supplementary Material

Microbial communities of seawater and coastal soil of Russian Arctic region and their potential for bioremediation from hydrocarbon pollutants

Ekaterina M. Semenova [1], Tamara L. Babich [1], Diyana S. Sokolova [1], Alexey P. Ershov[1], Yeva I. Raievska [1], Salimat K. Bidzhieva [1], Alexey L. Stepanov [2], Maria V. Korneykova [3,4] , Vladimir A. Myazin [3] and Tamara N. Nazina [1,*]

[1] Winogradsky Institute of Microbiology, Research Center of Biotechnology of the Russian Academy of Sciences, Moscow, 119071, Russia; semenova_inmi@mail.ru (E.M.S.); microb101@yandex.ru (T.L.B.); sokolovadiyana@gmail.com (D.S.S.); e.alexey.mail@inmi.ru (A.P.E.); raievska.yeva@mail.ru (Y.I.R); salima.bidjieva@gmail.com (S.K.B.), nazina@inmi.ru (T.N.N.)

[2] Soil Department, Moscow State University, Moscow, 119991, Russia; stepanov_aleksey@mail.ru (A.L.S.)

[3] Institute of North Industrial Ecology Problems - Subdivision of the Federal Research Centre "Kola Science Centre of Russian Academy of Science", 184209 Apatity, Russia; myazinv@mail.ru (V.A.M.)

[4] Agrarian and Technological Institute, Peoples' Friendship University of Russia (RUDN University), Moscow, 117198, Russia; korneykova.maria@mail.ru (M.V.K.)

* Correspondence: nazina@inmi.ru; Tel.: +7-499-135-0341 (T.N.N.)

* Correspondence: korneykova.maria@mail.ru; Tel.: +7-921-288-8830 (M.V.K.)

Table S1. The content of alcohols and lower fatty acids (mg·l^{-1} sea water or mg·g^{-1} soil) in the studied samples of seawater, littoral sandy mud, and coastal soil collected at the Murmansk region.

Sample number	Location	Sample	Ethanol	Acetate	Σ(C_3-C_5 VFA)
1_M21	Belokamenka	Water	0	121	178
6_M21		Littoral ground	11	56	11
7_M21		Coastal soil	8	41	12
2_M21	Roslyakovo	Water	0	90	10
10_M21		Littoral ground	8	18	59
9_M21		Coastal soil	25	18	11
11_M21	Kola	Littoral ground	23	18	6
3_M21	Pechenga	Littoral ground	0	32	7

Table S2. The number of the 16S rRNA gene sequences and operational taxonomic units (OTUs), coverage and diversity indices in libraries of bacterial communities of the seawater (1_M21 and 2_M21), littoral sandy mud (3_M21, 6_M21, and 10_M21) and coastal soil (7_M21 and 9_M21) samples.

Sample	Number of total reads	Number of OTU	CHAO index	Shannon diversity index	Good's coverage (%)
1_M21	797	145	171.7	3.5	0.9
2_M21	298	146	176.1	4.6	0.7
3_M21	3675	1221	575.3	6.1	0.8
6_M21	3915	1305	583.5	6.5	0.8
10_M21	4085	6257	723.8	7.3	0.7
7_M21	6257	1446	510.4	6.2	0.9
9_M21	6043	1124	453.6	5.0	0.9

Table S3. Heatmap showing major bacterial classes proportions based on 16S rRNA gene amplicon sequencing in samples of seawater, littoral sandy mud, and coastal soil.

Class	1_M21	6_M21	7_M21	2_M21	9_M21	10_M21	3_M21
Acidimicrobiia	0.1	6.7	3.0	0.7	1.7	6.6	8.5
Actinomycetes	1.9	4.8	8.5	7.1	19.6	5.7	9.7
Alphaproteobacteria	7.5	16.5	36.5	13.6	12.5	17.6	28.0
Anaerolineae	0.1	0.4	0.4	0.3	0.4	0.1	1.9
Bacilli	4.1	0.8	0.1	7.4	0.6	0.8	0.2
Bacteroidia	2.6	9.8	2.6	5.2	2.3	9.0	1.6
Blastocatellia	0.0	3.5	0.4	0.0	0.2	0.8	0.0
Chlamydiia	0.3	0.5	0.9	0.7	1.2	0.5	0.3
Chloroflexia	0.0	0.2	0.3	0.0	0.9	0.4	2.7
Clostridia	2.2	0.1	0.2	19.2	2.2	1.5	1.2
Coriobacteriia	0.1	0.0	0.0	3.2	0.0	0.0	0.6
Cyanophyceae	0.2	4.9	0.3	0.3	0.2	0.6	11.9
Deltaproteobacteria	0.9	1.3	0.7	2.7	1.6	1.8	6.2
Epsilonproteobacteria	0.8	0.0	0.0	3.9	0.0	0.1	0.4
Gammaproteobacteria	70.2	30.5	20.4	29.1	40.3	31.4	8.2
Gemmatimonadetes	0.2	0.5	1.0	0.0	0.4	0.3	0.2
Melainabacteria	1.2	0.1	0.1	0.0	0.1	0.1	0.0
Negativicutes	0.0	0.0	0.0	1.5	0.0	0.0	0.0
Omnitrophica	0.0	0.0	0.0	2.2	0.0	0.0	0.0
Planctomycetia	1.6	8.9	7.9	0.5	2.2	8.0	6.3
Rhodothermia	0.0	2.0	0.0	0.0	0.0	1.0	0.1
Thermoanaerobaculia	0.0	1.2	0.5	0.0	0.0	2.5	0.2
Thermoleophilia	0.3	0.6	4.9	0.2	6.0	1.2	5.0
Verrucomicrobiae	0.2	1.6	3.3	0.2	1.3	0.9	2.6
Vicinamibacteria	0.1	0.3	1.2	0.0	0.7	1.0	0.2
Other	5.4	4.8	6.9	2.0	5.3	7.9	3.9
Total	100.0	100.0	100.0	100.0	100.0	100.0	100.0

Table S4. Contribution of key bacteria to the metabolism of nitrogen compounds in the studied microbial communities (%).

Bacteria	1_M21	6_M21	7_M21	2_M21	9_M21	10_M21	3_M21
Pseudomonas	6.8	6.4	27.6	14.0	81.3	3.1	0.0
Serratia	28.1	0.7	0.1	0.0	5.3	1.0	0.0
Blastopirellula	0.0	27.5	26.9	0.0	0.0	12.4	51.3
Yersinia	24.8	0.0	0.1	0.0	3.1	0.0	0.0
Oxalobacteraceae	22.4	0.1	0.0	0.0	0.1	0.0	0.0
Rhodobacteraceae	1.7	1.5	1.9	24.1	0.0	15.6	0.2
Nocardioides	0.0	4.8	14.0	0.0	3.3	6.5	14.7
Mycobacterium	0.2	11.1	1.5	3.9	1.1	29.5	2.8
Trichococcus	0.0	3.7	16.0	13.6	0.5	1.8	0.1
Rhodanobacter	4.0	0.3	2.4	0.0	0.4	0.0	16.3
Acidovorax	2.2	0.7	0.0	17.3	0.2	0.0	0.0
Gammaproteobacteria	0.0	5.7	0.8	3.6	0.0	15.1	8.0
Glaciecola	3.7	0.0	0.0	11.0	0.0	0.4	0.0
Sphingomonas	0.8	0.0	7.0	7.0	1.5	0.7	0.1
Solirubrobacterales	0.1	11.8	0.9	0.0	2.1	6.6	2.4
Maribacter	0.0	13.5	0.9	0.0	0.0	4.1	2.8
Herminiimonas	4.8	0.2	0.0	0.0	0.0	0.0	0.0
Actinobacteria	0.2	3.7	0.0	5.7	0.1	1.1	1.2
Chloroflexi	0.0	8.2	0.1	0.0	0.9	2.2	0.1

Table S5. Contribution of key bacteria to the metabolism of sulfur compounds in the studied microbial communities (%).

Bacteria	1_M21	6_M21	7_M21	2_M21	9_M21	10_M21	3_M21
Pseudomonas	6.2	6.1	27.5	13.1	78.1	2.8	0.0
Serratia	29.6	0.8	0.1	0.0	6.0	1.1	0.0
Yersinia	30.7	0.0	0.1	0.0	4.1	0.0	0.0
Blastopirellula	0.0	23.4	23.9	0.0	0.0	10.2	47.3
Oxalobacteraceae	17.5	0.1	0.0	0.0	0.1	0.0	0.0
Nocardioides	0.0	5.3	16.2	0.0	3.7	6.9	17.6
Rhodobacteraceae	1.6	1.6	2.0	24.4	0.0	15.5	0.2
Mycobacterium	0.2	12.0	1.7	4.1	1.2	30.9	3.3
Trichococcus	0.0	3.3	14.7	11.7	0.4	1.5	0.1
Solirubrobacterales	0.2	15.7	1.2	0.0	2.9	8.4	3.5
Acidovorax	2.1	0.7	0.0	17.2	0.2	0.0	0.0
Glaciecola	4.0	0.0	0.0	12.1	0.0	0.4	0.0
Sphingomonas	0.9	0.0	8.8	8.2	1.8	0.8	0.1
Rhodanobacter	3.3	0.2	2.2	0.0	0.4	0.0	15.4
Gammaproteobacteria	0.0	5.6	0.8	3.5	0.0	14.4	8.5
Maribacter	0.0	11.6	0.8	0.0	0.0	3.4	2.6
Actinobacteria	0.2	3.8	0.0	5.7	0.1	1.1	1.3
Herminiimonas	3.4	0.1	0.0	0.0	0.0	0.0	0.0
Chloroflexi	0.0	9.6	0.1	0.0	1.0	2.4	0.1

Table S6. Contribution of key bacteria to benzoate metabolism in the studied microbial communities (%).

Bacteria	1_M21	6_M21	7_M21	2_M21	9_M21	10_M21	3_M21
Pseudomonas	7.7	7.4	31.7	12.7	78.4	2.8	0.0
Serratia	27.7	0.7	0.1	0.0	4.5	0.8	0.0
Oxalobacteraceae	27.8	0.2	0.0	0.0	0.1	0.0	0.0
Nocardioides	0.0	8.8	25.9	0.0	5.1	9.5	33.8
Rhodobacteraceae	2.3	2.1	2.6	26.4	0.0	17.1	0.4
Sphingomonas	2.1	0.0	18.3	14.4	3.4	1.4	0.3
Yersinia	16.6	0.0	0.0	0.0	1.8	0.0	0.0
Mycobacterium	0.3	16.4	2.2	4.5	1.4	34.5	5.2
Solirubrobacterales	0.3	26.4	2.0	0.0	4.0	11.6	6.7
Acidovorax	2.9	1.0	0.0	18.0	0.2	0.0	0.0
Glaciecola	5.0	0.0	0.0	11.9	0.0	0.4	0.0
Blastopirellula	0.0	9.0	8.8	0.0	0.0	3.2	21.0
Gammaproteobacteria	0.0	6.4	0.9	3.2	0.0	13.6	11.3
Rhodanobacter	3.3	0.2	2.1	0.0	0.3	0.0	17.1
Actinobacteria	0.3	4.8	0.0	5.8	0.1	1.2	1.9
Herminiimonas	3.6	0.1	0.0	0.0	0.0	0.0	0.0
Trichococcus	0.0	1.1	4.8	3.2	0.1	0.4	0.1
Maribacter	0.0	8.3	0.6	0.0	0.0	2.0	2.1
Chloroflexi	0.0	7.1	0.1	0.0	0.6	1.5	0.1

Table S7. Contribution of key bacteria to the metabolism of polycyclic aromatic hydrocarbons in the studied microbial communities (%).

Bacteria	1_M21	6_M21	7_M21	2_M21	9_M21	10_M21	3_M21
Pseudomonas	10.6	12.0	35.0	17.6	84.7	4.8	0.0
Serratia	36.3	9.4	11.3	0.0	4.6	1.3	0.0
Rhodobacteraceae	3.2	3.5	2.9	37.1	0.0	29.9	0.8
Sphingomonas	2.1	0.0	28.4	14.2	2.6	3.2	6.3
Oxalobacteraceae	23.0	0.2	0.0	0.0	0.0	0.0	0.0
Nocardioides	0.0	7.1	14.2	0.0	2.8	8.1	35.1
Yersinia	14.8	0.0	0.0	0.0	1.3	0.0	0.0
Solirubrobacterales	0.3	27.8	1.4	0.0	2.8	13.0	9.1
Acidovorax	2.2	0.9	0.0	14.1	0.1	0.0	0.0
Mycobacterium	0.2	8.9	0.8	2.1	0.5	19.8	3.6
Glaciecola	3.5	0.0	0.0	8.5	0.0	0.4	0.0
Gammaproteobacteria	0.0	5.5	0.5	2.3	0.0	12.3	12.4
Blastopirellula	0.0	6.4	4.3	0.0	0.0	2.4	19.2
Rhodanobacter	1.3	0.1	0.7	0.0	0.1	0.0	10.3
Actinobacteria	0.2	3.1	0.0	4.0	0.1	1.2	0.2
Maribacter	0.0	8.9	0.4	0.0	0.0	2.3	2.9
Herminiimonas	2.3	0.1	0.0	0.0	0.0	0.0	0.0
Chloroflexi	0.0	6.0	0.0	0.0	0.4	1.3	0.1

Table S8. Contribution of key bacteria to the degradation of fatty acids in the studied microbial communities (%).

Bacteria	1_M21	6_M21	7_M21	2_M21	9_M21	10_M21	3_M21
Pseudomonas	7.2	5.2	25.9	10.4	74.1	2.0	0.0
Nocardioides	0.0	9.8	32.9	0.0	7.5	10.6	37.7
Oxalobacteraceae	29.7	0.1	0.0	0.0	0.1	0.0	0.0
Serratia	23.1	0.5	0.1	0.0	3.8	0.5	0.0
Mycobacterium	0.5	17.7	2.7	5.6	2.0	37.7	5.7
Rhodobacteraceae	2.7	1.9	2.7	26.7	0.0	15.3	0.3
Solirubrobacterales	0.4	28.2	2.4	0.0	5.7	12.5	7.2
Yersinia	16.6	0.0	0.0	0.0	1.8	0.0	0.0
Sphingomonas	1.9	0.0	14.6	11.4	3.1	1.0	0.2
Glaciecola	6.9	0.0	0.0	14.5	0.0	0.5	0.0
Blastopirellula	0.0	9.1	10.2	0.0	0.0	3.3	21.4
Acidovorax	3.2	0.8	0.0	17.5	0.2	0.0	0.0
Gammaproteobacteria	0.0	5.4	0.8	3.1	0.0	11.6	9.6
Rhodanobacter	3.5	0.2	1.9	0.0	0.3	0.0	13.8
Actinobacteria	0.5	5.2	0.0	7.3	0.2	1.3	2.1
Trichococcus	0.0	1.0	5.1	3.4	0.2	0.4	0.0
Herminiimonas	3.9	0.1	0.0	0.0	0.0	0.0	0.0
Chloroflexi	0.0	8.0	0.1	0.0	1.0	1.7	0.1
Maribacter	0.0	6.8	0.5	0.0	0.0	1.7	1.8

Table S9. Contribution of key bacteria to methane metabolism in the studied microbial communities (%).

Bacteria	1_M21	6_M21	7_M21	2_M21	9_M21	10_M21	3_M21
Pseudomonas	4.8	4.3	21.7	10.0	72.0	2.1	0.0
Serratia	31.4	0.8	0.1	0.0	7.6	1.1	0.0
Yersinia	26.9	0.0	0.1	0.0	4.3	0.0	0.0
Blastopirellula	0.0	22.2	25.7	0.0	0.0	10.1	45.7
Oxalobacteraceae	19.3	0.1	0.0	0.0	0.1	0.0	0.0
Rhodobacteraceae	2.2	1.9	2.7	31.6	0.0	19.1	0.3
Nocardioides	0.0	5.5	18.9	0.0	5.0	7.5	18.4
Solirubrobacterales	0.2	18.5	1.6	0.0	4.4	10.3	4.1
Mycobacterium	0.2	8.5	1.3	3.2	1.2	22.7	2.4
Trichococcus	0.0	2.7	13.9	10.7	0.5	1.3	0.1
Gammaproteobacteria	0.0	6.1	1.0	4.1	0.0	16.3	9.4
Rhodanobacter	3.5	0.2	2.4	0.0	0.5	0.0	15.0
Sphingomonas	1.0	0.0	9.5	8.6	2.3	0.8	0.1
Glaciecola	3.8	0.0	0.0	11.4	0.0	0.4	0.0
Acidovorax	1.6	0.5	0.0	12.9	0.2	0.0	0.0
Actinobacteria	0.3	4.5	0.0	7.5	0.2	1.4	1.6
Herminiimonas	4.8	0.1	0.0	0.0	0.0	0.0	0.0
Chloroflexi	0.0	12.3	0.1	0.0	1.7	3.3	0.2
Maribacter	0.0	11.9	1.0	0.0	0.0	3.7	2.7

Table S10. Taxonomic affiliation of pure bacterial cultures isolated from samples of seawater, littoral soil and coastal soil.

Strain	GenBank number	Closest type strain according to 16S rRNA gene, acc. no.	16S rRNA gene similarity, %	Isolation source
M3-1	MW853692	*Aeromonas salmonicida subsp. pectinolytica* 34mel, AF134065	99.8	3_M21
M6-13	MZ620650	*Oceanisphaera marina* YM319, NR_157015	99.8	6_M21
M6-14	MZ620656	*Oceanisphaera marina* YM319, NR_157015	99.6	6_M21
M10-21	MZ620680	*Paeniglutamicibacter psychrophenolicus* AG31, NR_027226.1	99.7	10_M21
M9-2	MW854008	*Pseudomonas prosekii* AN/28/1, JN814372	99.9	9_M21
M9-22	MZ620679	*Pseudomonas baetica* a390,FM201274	99.7	9_M21
M7-26	MZ620683	*Pseudomonas baetica* a390,FM201274	99.8	7_M21
M6-6	MW853771	*Pseudomonas brenneri* BD11-00181, KU647658	99.6	6_M21
M3-10	MW853765	*Pseudomonas guineae* M8, NR_042607	99.8	3_M21
M11-25	OM273845	*Pseudomonas kielensis* MBT-1, MW377589	99.9	11_M21
M11-3	MW854025	*Pseudomonas leptonychotis* CCM 8849, MK104124	99.9	11_M21
M7-27	MZ620702	*Pseudomonas protegens* CHA0, MN749571	99.7	7_M21
M9-9	MZ636810	*Pseudomonas silesiensis* A3, NR_156815	100	9_M21
M6-11	MW854024	*Rhodococcus fascians* ATCC 12974, X81930	100	6_M21
M6-12	MW854029	*Rhodococcus fascians* ATCC 12974, X81930	99.6	6_M21
M7-8	MW853833	*Rhodococcus erythropolis* JCM 15477, MK424306	100	7_M21
M2-15	MZ620649	*Rhodococcus erythropolis* JCM 15477, MK424306	100	2_M21
M7-5	MW853791	*Serratia myotis* 12, KJ739884	100	7_M21
M3-18	MZ620714	*Shewanella livingstonensis* NF22, NR_025443	100	3_M21

Table S11. Surface tension (ST) and interfacial tension (IT) of the culture liquid of aerobic bacteria grown in a medium with crude oil at 10 °C for 30 days.

Strain	ST, mN/m	IT, mN/m
Aeromonas salmonicida M3-1	50.6	3.9
Oceanisphaera marina M6-14	54.8	27.3
Pseudomonas baetica M9-22	51.0	27.3
Pseudomonas brenneri M6-6	52.1	10.8
Pseudomonas guinea M3-10	59.9	39.8
Pseudomonas kielensis M11-25	51.0	26.9
Pseudomonas leptonychotis M11-3	54.2	19.2
Serratia myotis M7-5	65.6	41.8
Pseudomonas protegens M7-27	52.2	29.8
Pseudomonas silesiensis M9-9	52.8	30.1
Rhodococcus erythropolis M7-8	46.3	5.2
Shewanella livingstonensis M3-18	53.8	31.5
Sterile medium	60.4	28.1

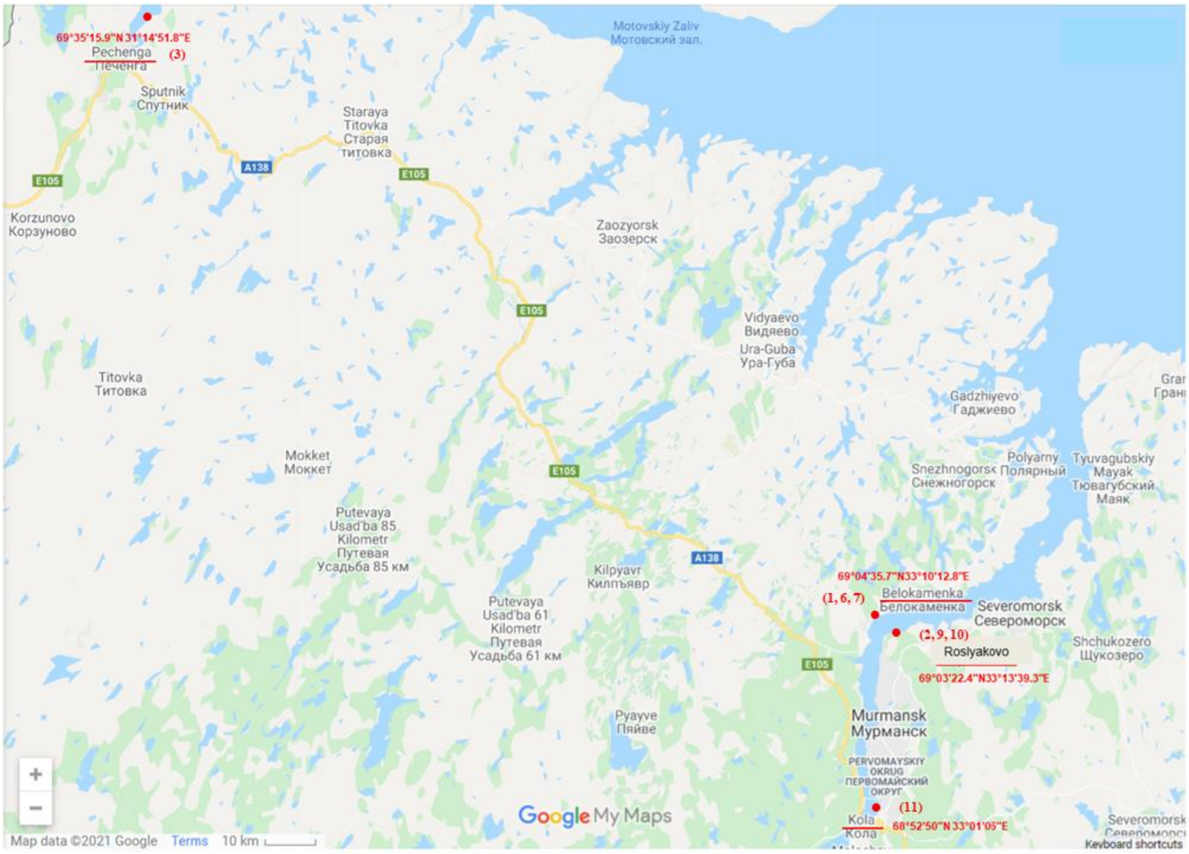

Figure S1. Sampling sites at the Murmansk region.

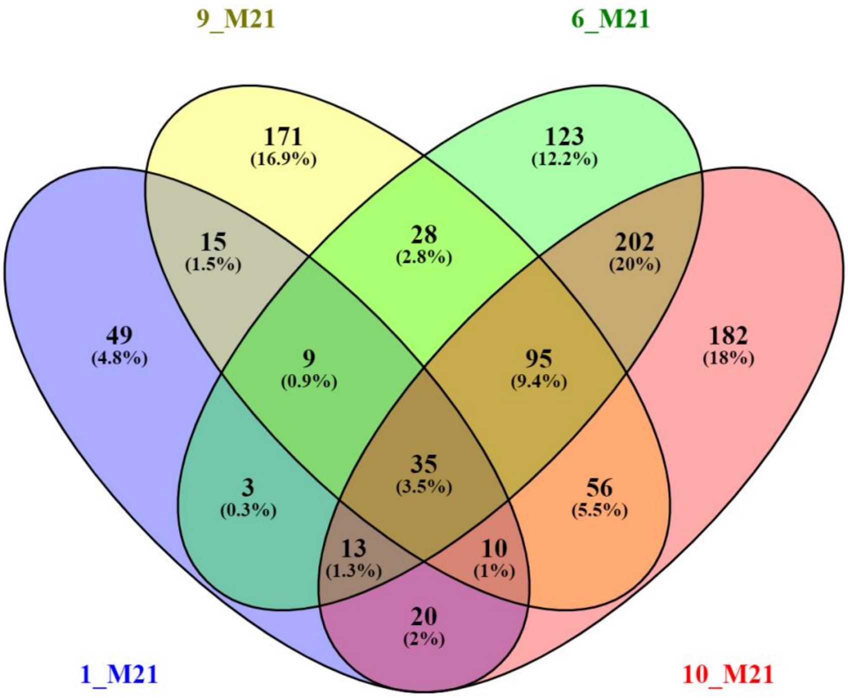

Figure S2. Venn diagram of the OTUs in the seawater (1_M21), littoral sandy mud (6_M21 and 10_M21) and coastal soil (9_M21) samples.

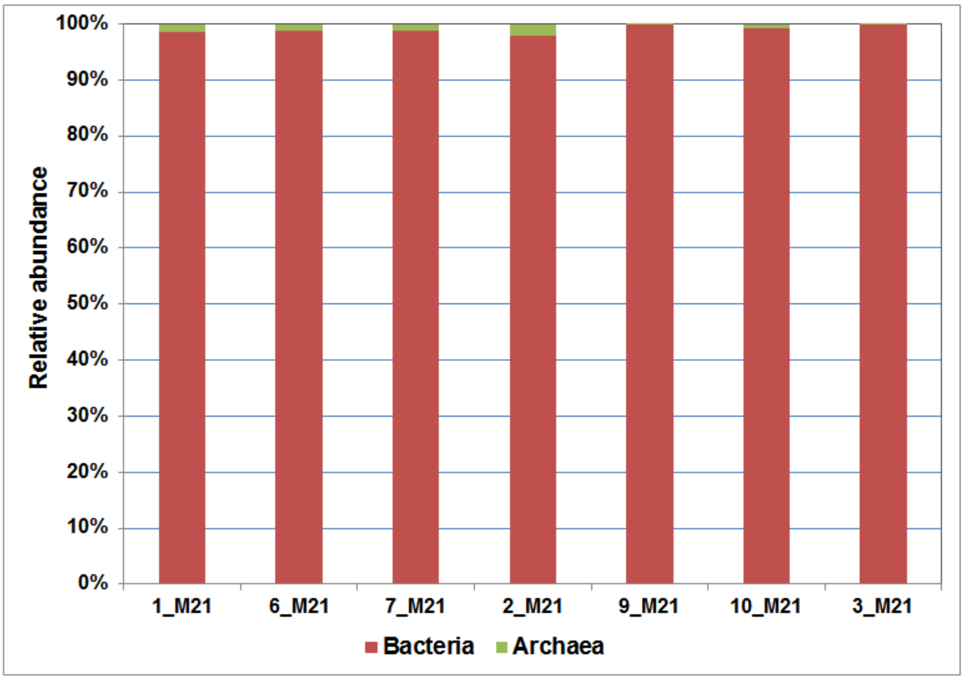

Figure S3. Relative abundance of the 16S rRNA gene fragments of the Bacteria and Archaea in the libraries of prokaryotic communities in samples of seawater (1_M21 and 2_M21), of littoral soil (3_M21, 6_M21, and 10_M21), and of coastal soil (7_M21 and 9_M21) collected at the Murmansk region.

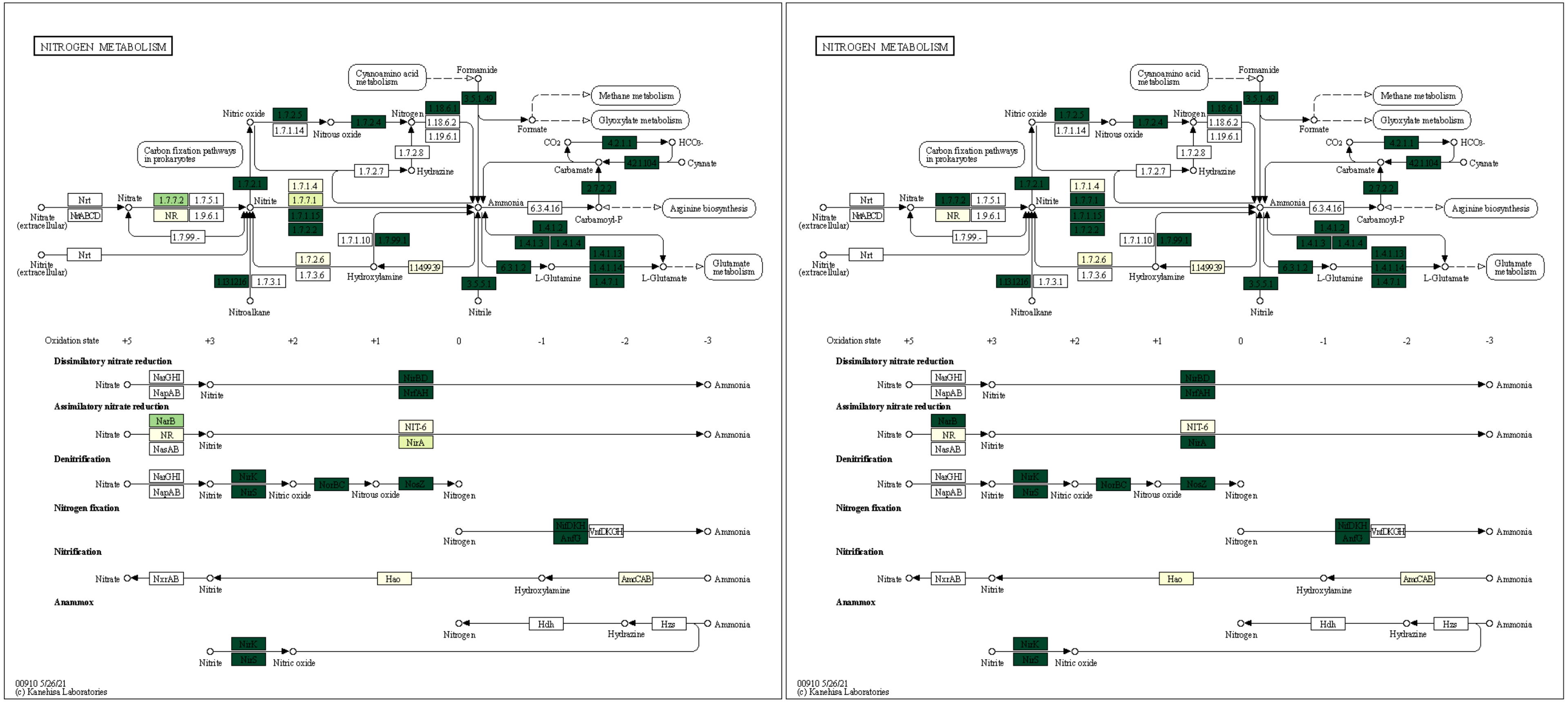

Figure S4. Predicted enzyme profiles for "Nitrogen metabolism" pathway (KO00910) in microorganisms of the seawater 1_M21 (left) and littoral soil 3_M21 (right) samples.

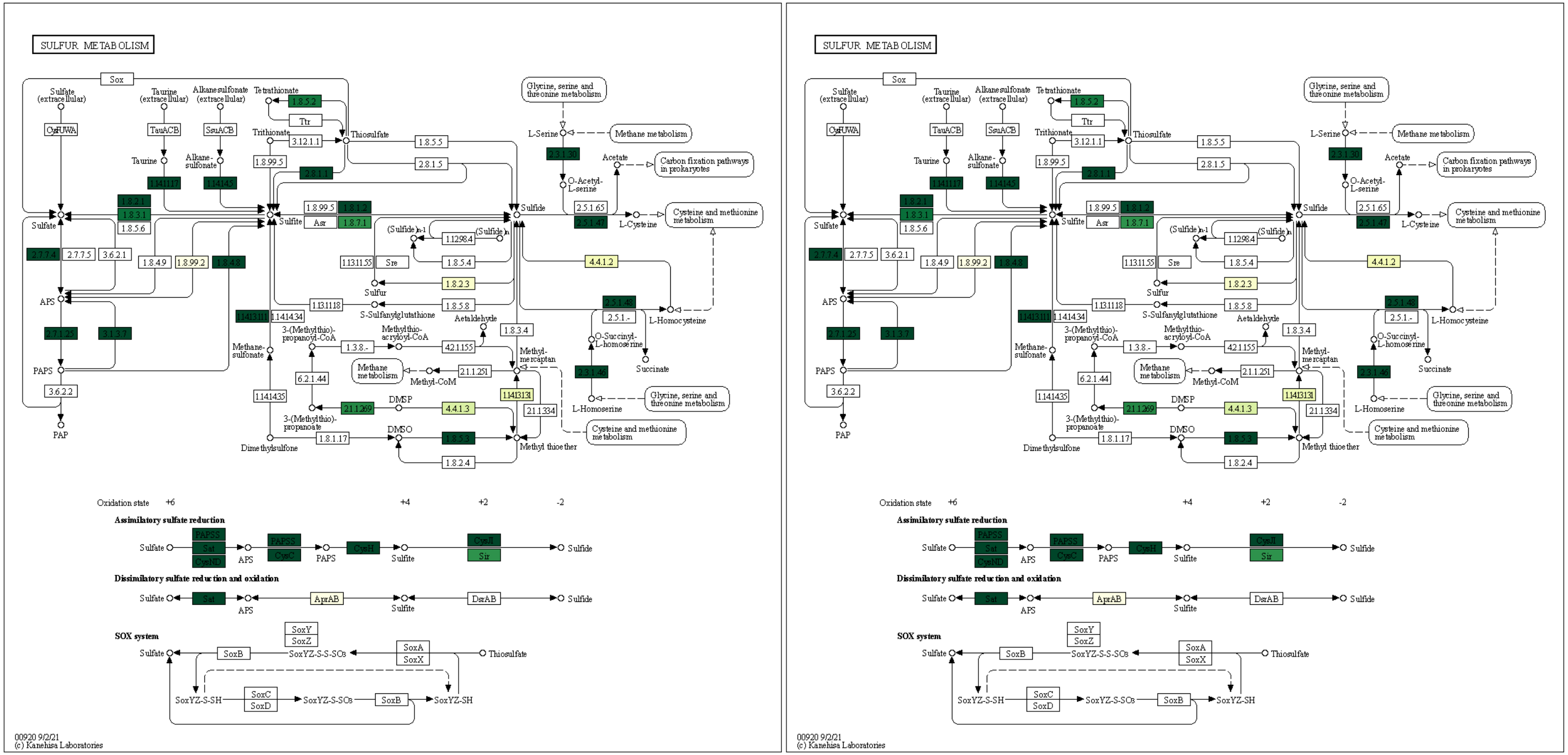

Figure S5. Predicted enzyme profiles for “Sulfur metabolism” pathway (KO00920) in microorganisms of the seawater 1_M21 (left) and littoral soil 3_M21 (right) samples.

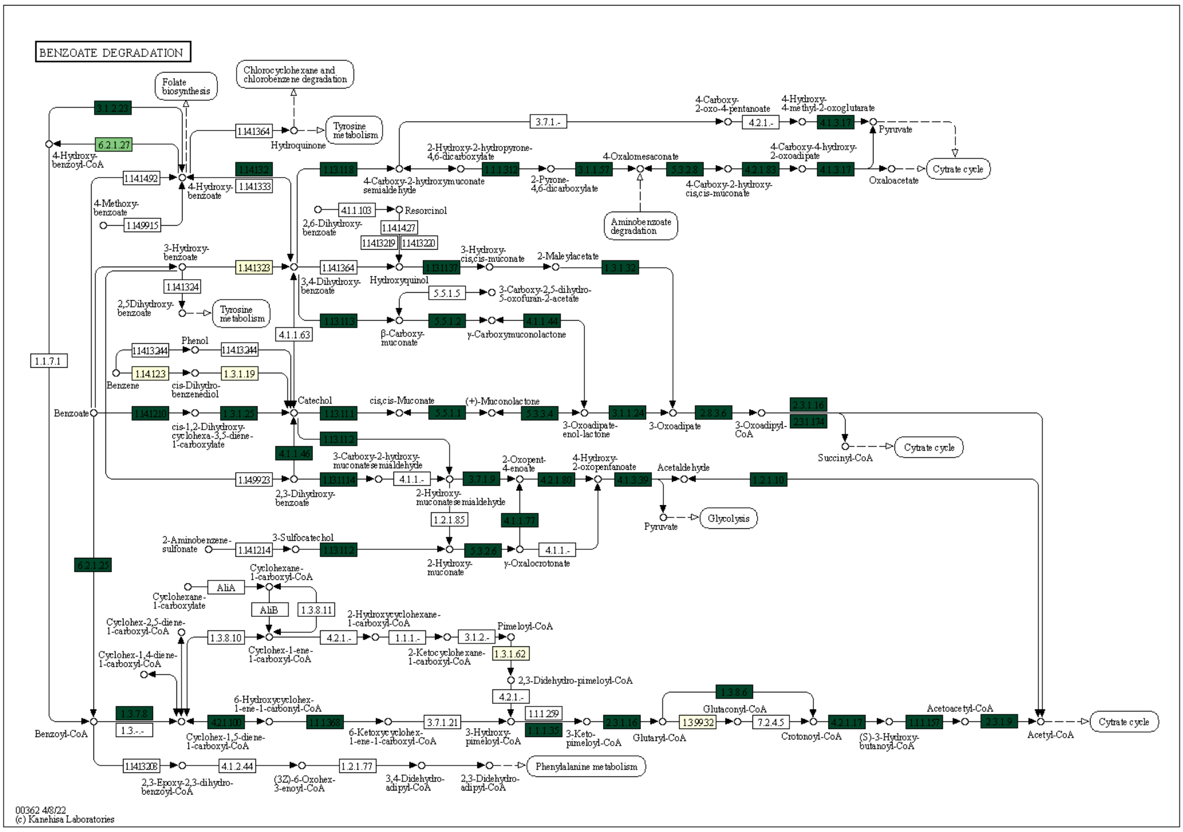

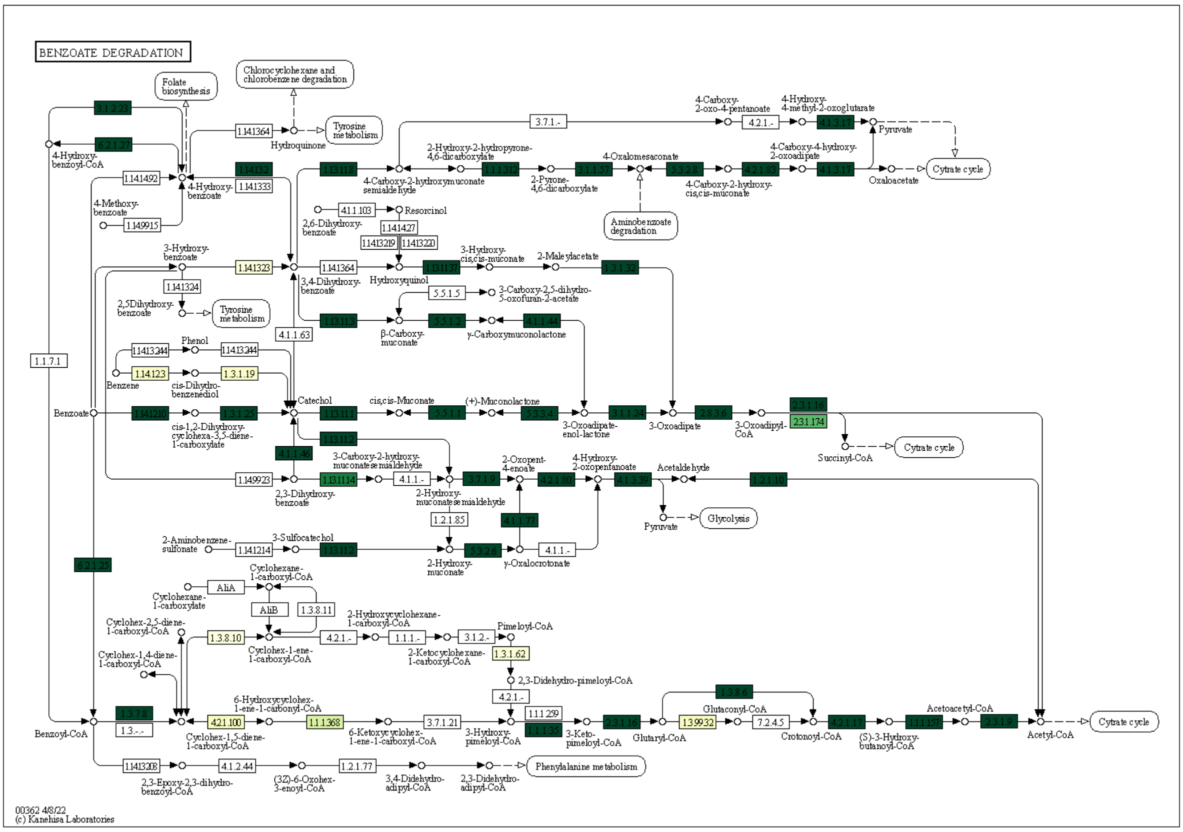

Figure S6. Predicted enzyme profiles for "Benzoate degradation" pathway (KO00362) in microorganisms of the seawater 1_M21 (upper) and littoral soil 3_M21 (lower) samples.

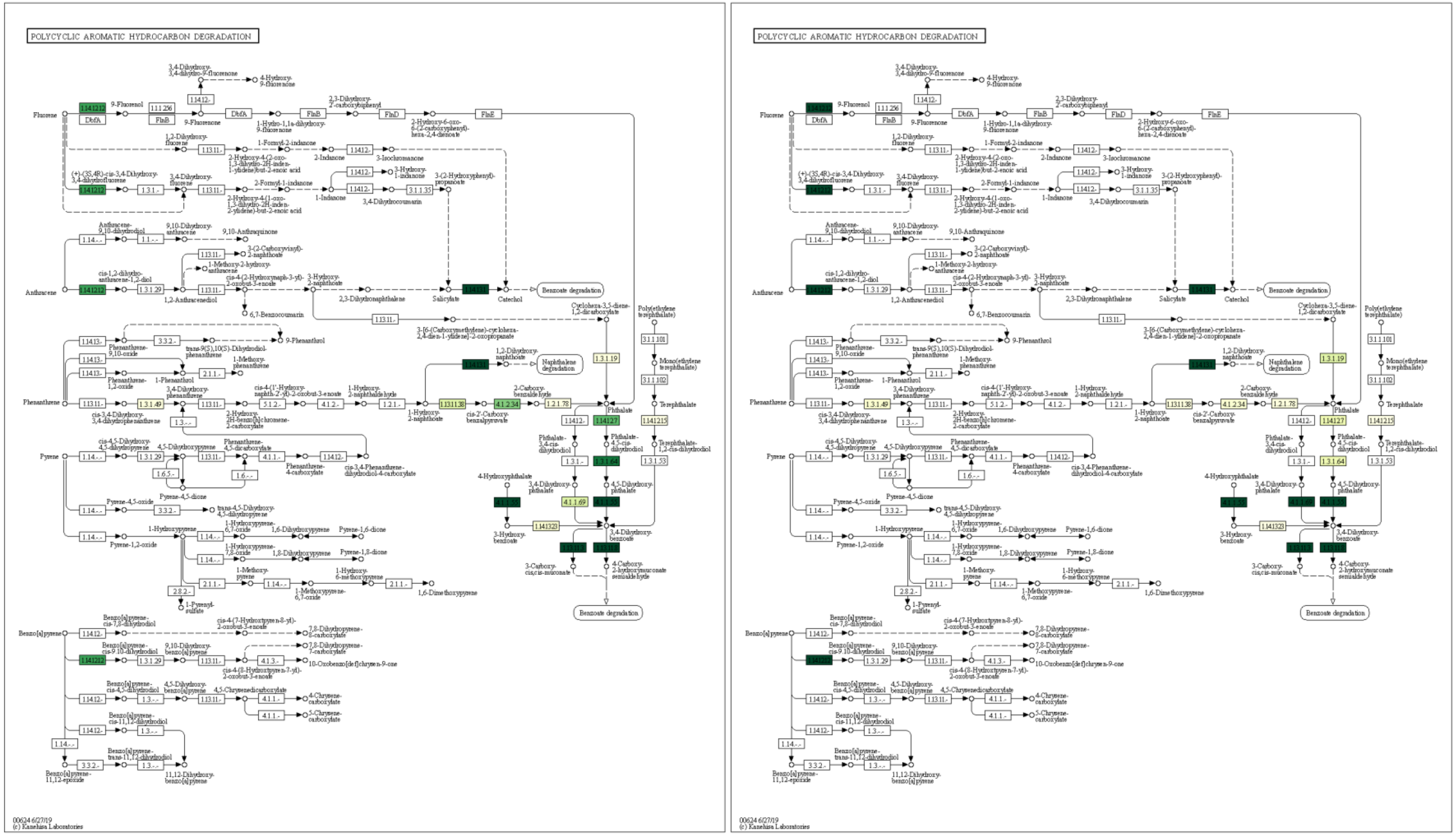

Figure S7. Predicted enzyme profiles for "Polycyclic aromatic hydrocarbon degradation" pathway (KO00624) in microorganisms of the seawater 1_M21 (left) and coastal soil 9_M21 (right) samples.

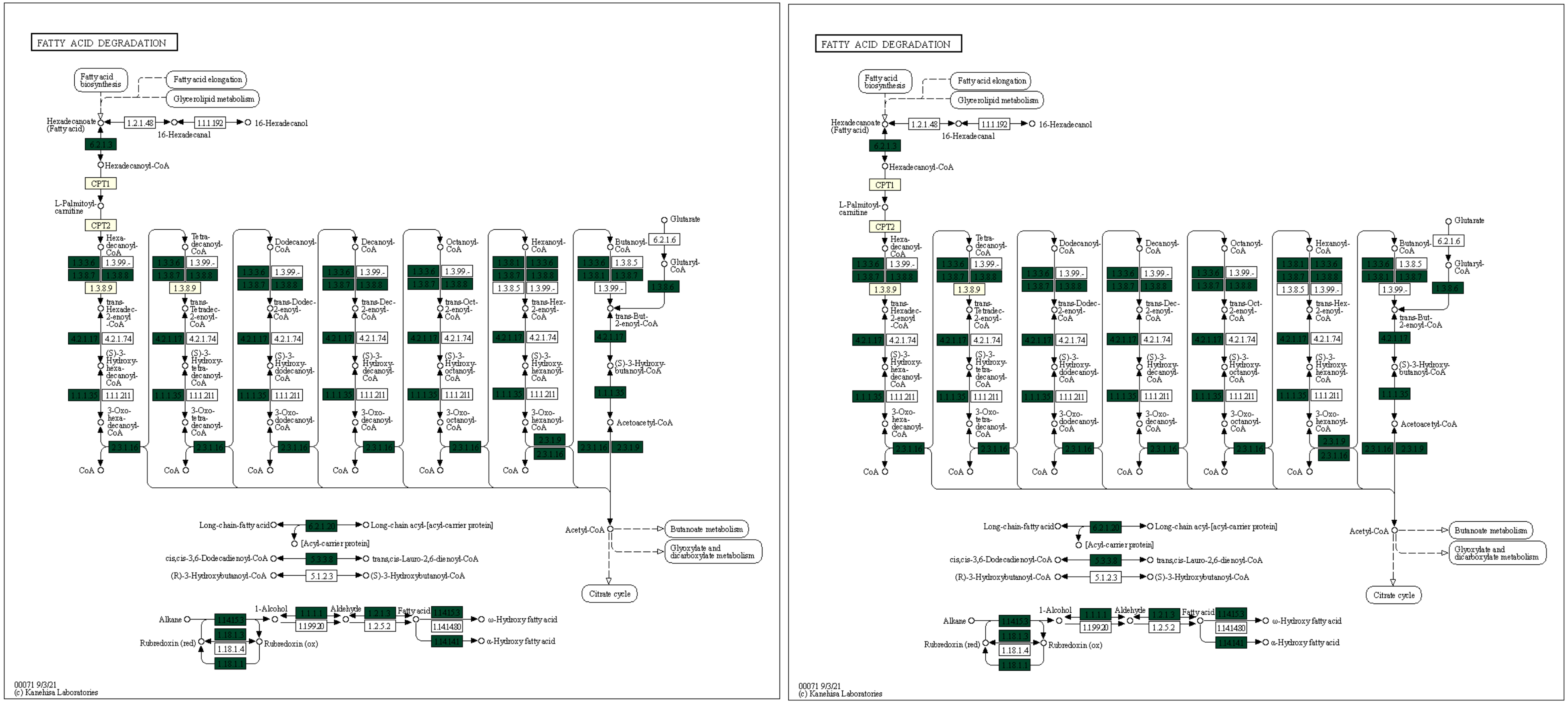

Figure S8. Predicted enzyme profiles for "Fatty acid degradation" pathway (KO00071) in microorganisms of the seawater 1_M21 (left) and coastal soil 9_M21 (right) samples.

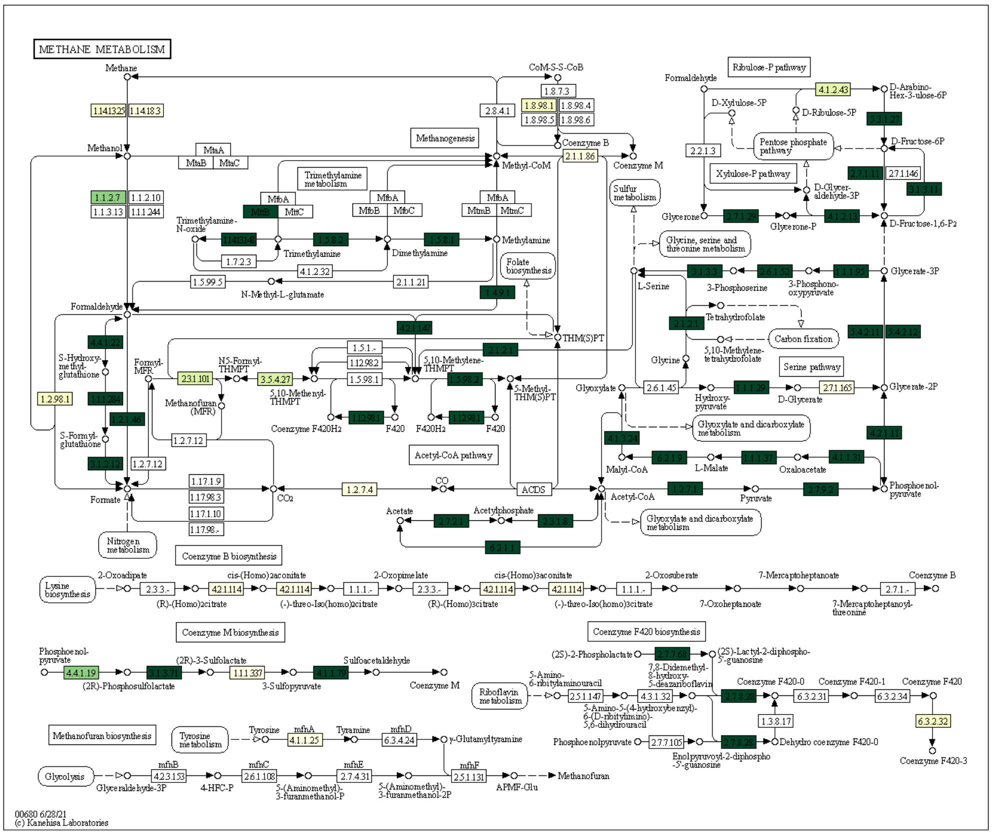

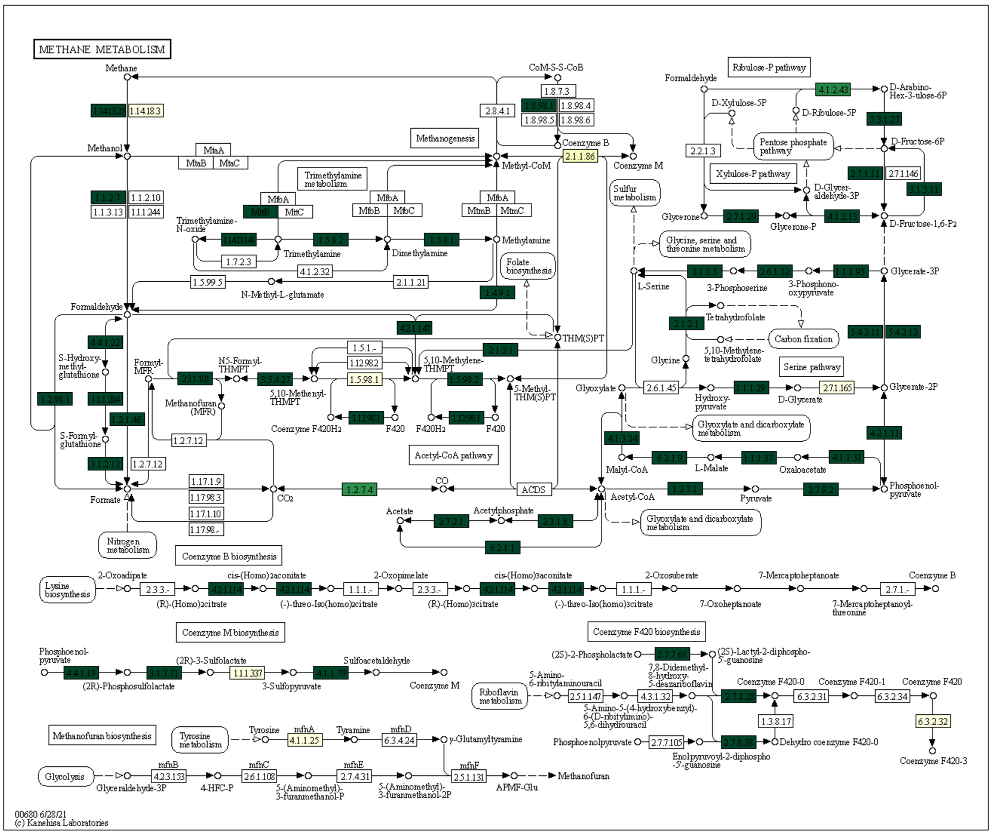

Figure S9. Predicted enzyme profile for "Methane metabolism" pathway (KO00680) in microorganisms of the seawater 1_M21 (upper) and littoral soil 3_M21 (lower) samples.

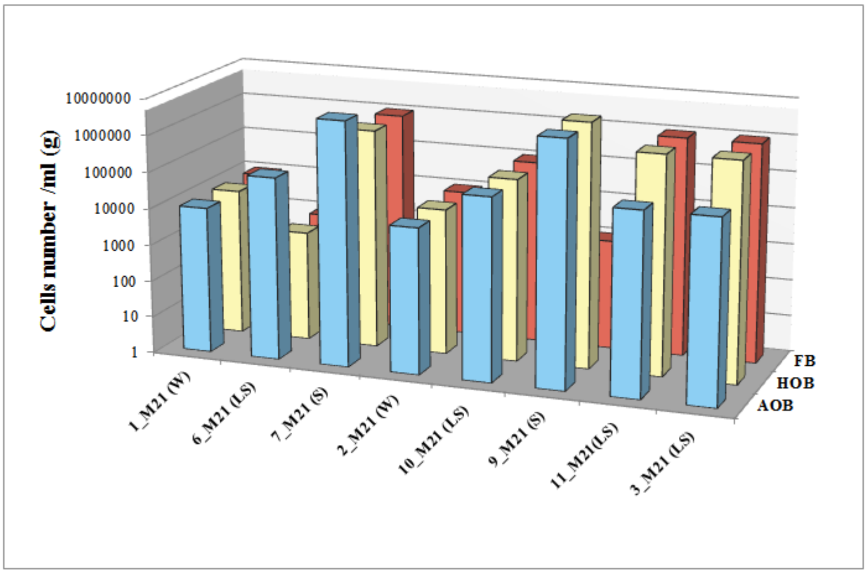

Figure S10. Cells number of aerobic organothrophic bacteria (AOB), hydrocarbon-oxidizing bacteria (HOB), and fermentative bacteria (FB) in samples of seawater (W), littoral sandy mud (LS), and coastal soil (S) collected at the Murmansk region.

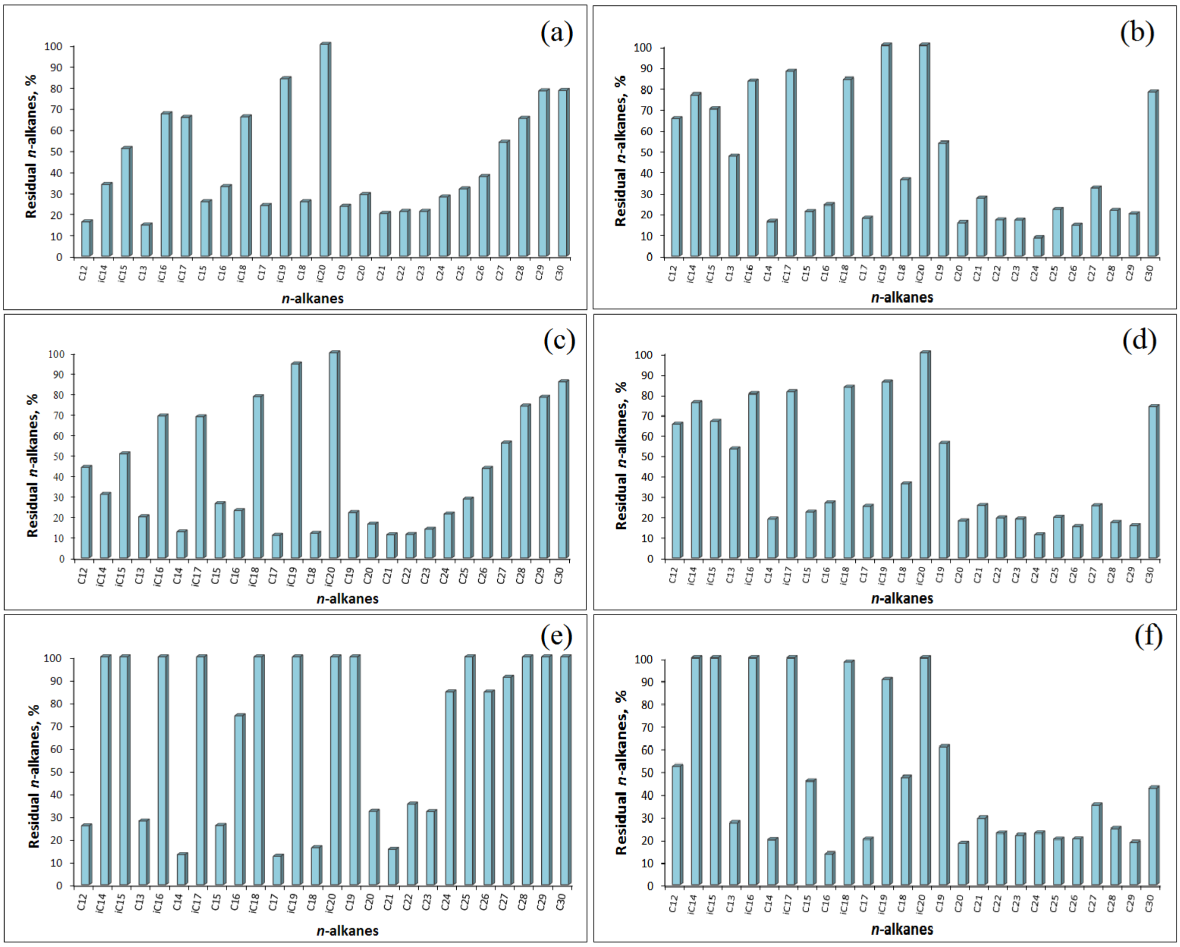

Figure S11. Residual content of *n*-alkanes in the aliphatic fraction of oil degraded by strains *Aeromonas salmonicida* M3-1 (a, b), *Pseudomonas brenneri* M6-6 (c, d), and *Rhodococcus erythropolis* M2-15 (e, f) in a liquid medium with oil for 30 days (a, c, e) and on sand with oil at 10 °C for 60 days (b, d, f).

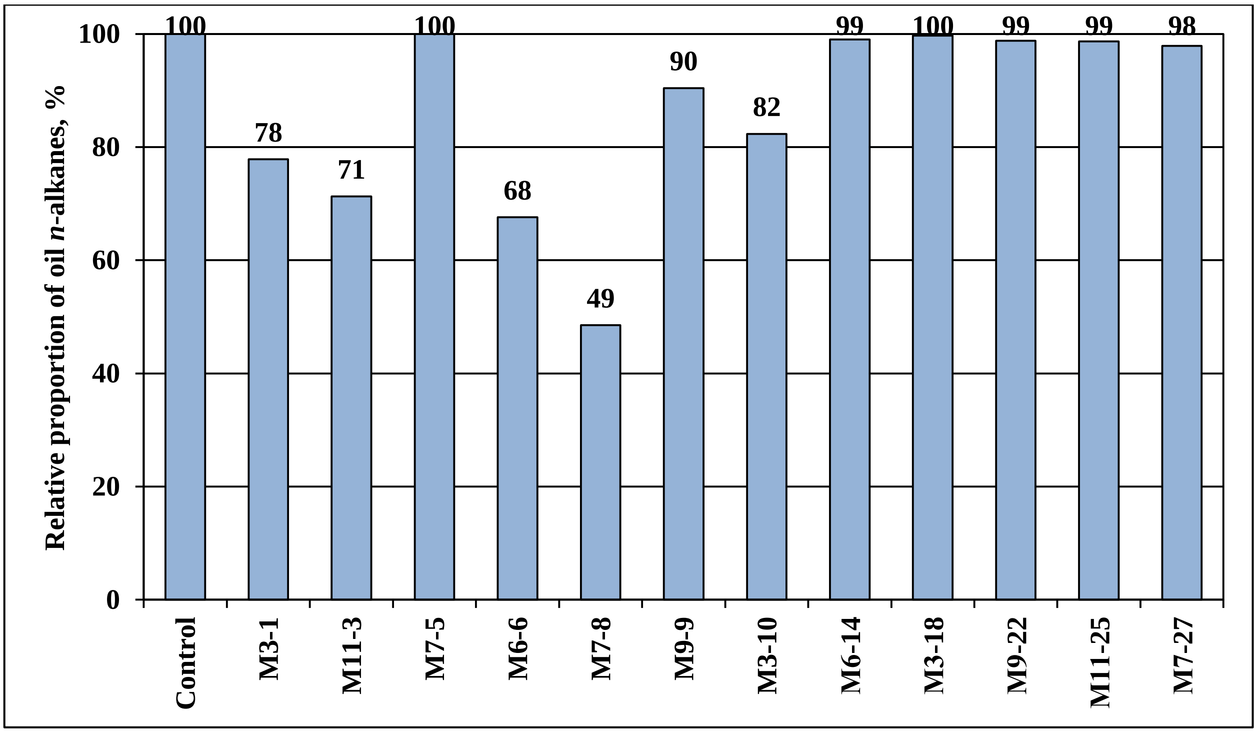

Figure S12. The utilization of crude oil *n*-alkanes by pure cultures *Aeromonas salmonicida* M3-1, *Pseudomonas leptonychotis* M11-3, *Serratia myotis* M7-5, *Pseudomonas brenneri* M6-6, *Rhodococcus erythropolis* M7-8, *Pseudomonas silesiensis* M9-9, *Pseudomonas guineae* M3-10, *Oceanisphaera marina* M6-14, *Shewanella livingstonensis* M3-18, *Pseudomonas baetica* M9-22, *Pseudomonas kielensis* M11-25, and *Pseudomonas protegens* M7-27.
